# Evaluating new species for aquaculture: A genomic dissection of growth in the New Zealand silver trevally (*Pseudocaranx georgianus*)

**DOI:** 10.1111/eva.13281

**Published:** 2021-07-30

**Authors:** Noemie Valenza‐Troubat, Elena Hilario, Sara Montanari, Peter Morrison‐Whittle, David Ashton, Peter Ritchie, Maren Wellenreuther

**Affiliations:** ^1^ The New Zealand Institute for Plant and Food Research Limited Nelson New Zealand; ^2^ The New Zealand Institute for Plant and Food Research Limited Auckland New Zealand; ^3^ The New Zealand Institute for Plant and Food Research Limited Motueka New Zealand; ^4^ School of Biological Sciences Victoria University of Wellington Wellington New Zealand; ^5^ School of Biological Sciences The University of Auckland Auckland New Zealand

**Keywords:** aquaculture, Carangidae, genetic diversity, growth traits, heritability

## Abstract

Aquaculture is the fastest‐growing food production sector worldwide, yet industry has been slow to implement genomic techniques as routine tools. Applying genomics to new breeding programmes can provide important information about pedigree structure and genetic diversity; key parameters for a successful long‐term breeding programme. It can also provide insights on potential gains for commercially important, yet complex, quantitative traits such as growth rate. Here we investigated a population of 1100 captive‐bred F_1_ silver trevally (*Pseudocaranx georgianus*), a promising new species for New Zealand aquaculture. We used whole‐genome information, coupled with image‐based phenotypic data collected over two years, to build the pedigree of the population, assess its genetic diversity, describe growth patterns of ten growth traits and estimate their genetic parameters. Successful parentage assignment of 664 F_1_ individuals showed that the pedigree consisted of a complex mixture of full‐ and half‐sib individuals, with skewed reproductive success among parents, especially in females. Growth patterns showed seasonal fluctuations (average increase across all traits of 27.3% in summer and only 7% in winter) and strong inter‐family differences. Heritability values for growth traits ranged from 0.27 to 0.76. Genetic and phenotypic correlations between traits were high and positive, ranging from 0.57 to 0.94 and 0.50 to 1.00 respectively. The implications of these findings are threefold: first, the best on‐growing conditions are in warmer months, where highest growth peaks can be achieved; second, size‐ and family‐based selection can be used as early selection criterion if pedigree structure and inbreeding risks are closely monitored; third, selection for body length results in concomitant increases in height and weight, traits of paramount importance for aquaculture. It is concluded that there is substantial potential for genetic improvement of economically important traits, suggesting that silver trevally is a promising species for selective breeding for enhanced growth.

## 1 INTRODUCTION

Aquaculture is the fastest growing food production sector worldwide (FAO, 2018). However, compared to terrestrial breeding programmes, the industry has been slow to implement the use of genomic information to inform selective decisions. This is despite the proven utility of genomics to provide insights into important commercial traits, such as faster growth and disease resistance (Gjedrem et al., 2012).

One of the most important and well‐studied traits in breeding programmes is growth rate (e.g. Ashton, Hilario et al., 2019; Ashton, Ritchie et al., 2019). This is because growth is easily quantified and often highly heritable and has a pronounced impact on commercial returns. In particular, improvements in growth rates reduce the overall time needed to raise individuals to market size, thereby speeding up the turnover of production stock and decreasing costs (Gjedrem, 2005; Ye et al., 2017). Usually, growth rate in animals is measured using either body weight or body length as a proxy, but as phenotyping methods improve, other traits such as width, girth and height are starting to be used to provide additional insights (Zenger et al., 2017). Image‐based phenotyping methods provide rapid and efficient measurements for multiple traits simultaneously. Moreover, datasets collected provide researchers with the opportunity to measure additional traits a posteriori, using computer vision based data mining at a later stage (e.g. body shape, health and colour) (Zenger et al., 2019).

While growth is of significant importance to aquaculture breeding programmes, genomic‐assisted selection for this trait is complicated by its typically polygenic basis (Wellenreuther & Hansson, 2016) and consequent requirement of large numbers of molecular markers. Genome‐wide markers have been used to identify loci associated with traits of importance and to guide selective breeding to efficiently increase phenotypic gains, but the application of genomic approaches has long been limited to model species or species of high economic value. Recently, the decrease in DNA sequencing and genotyping costs has meant that large numbers (e.g. thousands) of genome‐wide markers can be easily generated for almost any species, at a cost that can be afforded by research groups (Bernatchez et al., 2017). Reduced representations of the genome, such as in genotyping by sequencing (GBS) (Elshire et al., 2011), offer a quick and cost‐effective way of genotyping a large number of individuals. Whole genome sequencing (WGS) can be used to generate a reference genome, particularly when combined with long read sequencing techniques and additional techniques, such as Hi‐C.

Genomic insights can inform a number of breeding decisions. First, a potential barrier for new breeding programmes is the lack of prior knowledge on key parameters such as the relatedness among individuals and number of contributing parents (Falconer & Mackay, 1996). This information is particularly challenging to gather for aquatic species where the founding individuals might be caught from the wild and/or exhibit mass spawning reproductive behaviours. If not carefully monitored, lack of control can lead to inbreeding pressure and early loss of genetic diversity, which must be minimized for a successful long‐term breeding programme (Wang et al., 2002). Second, a useful parameter to asses is the selection potential of a trait. Estimates of trait heritability provide insights into the relative contribution of genetic variance compared with environmental variance. Knowing if the heritability of a trait is high (~0.9) or low (~0.1) will indicate how well the trait will respond to selection (Wray & Visscher, 2008). Broad‐sense heritability is defined as the ratio of the total genetic variance on the total phenotypic variance and includes effects such as dominance and epistasis, which do not respond to selection. Narrow‐sense heritability reflects the ratio of the additive genetic variance on the total phenotypic variance and represents the part that will respond to selection (Wray & Visscher, 2008). Third, another application of genomic tools for predicting the response of a trait to selection is the estimation of genetic correlations between two or more traits (Lynch, 1999). Because the genes that contribute to traits can be highly genetically correlated and co‐inherited, it is particularly important to know the magnitude and direction of these correlations when predicting breeding values. The improvement of multiple traits simultaneously may seem efficient but, because of unexpected correlations, it could result in the co‐selection of undesired phenotypes (Falconer & Mackay, 1996).

In Aotearoa New Zealand, aquaculture production relies almost exclusively on the farming of the three species: Greenshell™ mussels (*Perna canaliculus*); Pacific oysters (*Crassostrea gigas*); and only one finfish species, chinook/king salmon (*Oncorhynchus tshawytscha*), a species introduced from North America (Camara & Symonds, 2014; Davies et al., 2019). The paucity of species means that there is a strong need to add resilience to the sector by diversifying the range of species farmed. One possible candidate for commercial aquaculture is silver trevally (*Pseudocaranx georgianus*, Cuvier 1833) (referred to as trevally hereafter), a shoaling pelagic species found throughout the coastal waters of southern Australia and around New Zealand (Gomon et al., 2008; Smith‐Vaniz & Jelks, 2006). Indigenous Māori people have a strong cultural connection to trevally, where it is considered as taonga (i.e. has value or is treasured) and is referred to as araara. In many regions, trevally is a major component of recreational and commercial fisheries (MPI, 2021). *Pseudocaranx* is also a genus that appears to do well in farm‐like conditions, as demonstrated by the fact that other species in this genus have already been successfully developed for large‐scale commercial farming in Asia (FAO, 2018). Furthermore, this genus belongs to the carangid family, which contains other species with well‐established breeding programmes around the world, for example, for the yellowtail kingfish (*Seriola lalandi*).

In this study, we applied genomic tools to a recently assembled population of silver trevally (*Pseudocaranx georgianus*, Cuvier 1833). The goal of this study was to use genome‐wide data coupled with extensive phenotypic data to provide the first quantitative assessment of growth traits in a population of 1100 captive‐bred F_1_ trevally. Specifically, we used a mixture of WGS and GBS data to (1) reconstruct the molecular pedigree of the population, (2) calculate inbreeding values within each generation, (3) describe the growth patterns by adding image‐based phenotyping data collected over a period of two years to and finally (4) estimate the heritability and genetic correlations of ten growth traits to determine their potential for enhanced growth performance through a selective breeding programme.

## 2 METHODS

### 2.1 Populations studied and holding conditions

The trevally population studied consisted of an F_0_ wild broodstock (*n* = 22) and a captive reared F_1_ population (*n* = 1100). F_0_ individuals were originally captured during two net tows in February 2012 in the North Taranaki Bight (Lat. 3845267–Long.17420626 and Lat. 3851887–Long. 17419780). Live fish arrived two days later at the Wakefield Key Finfish Facility (formerly operated by The New Zealand Institute for Plant and Food Research Limited (PFR) in Nelson, New Zealand) and acclimated to a single 4400 L tank. Broodstock (remaining *n* = 19) were later transferred to the Maitai Finfish Facility (currently operated by PFR in Nelson, New Zealand) in 2014 and were acclimated to a single 13,000 L tank, where all research was subsequently carried out. The Finfish Facility receives ambient seawater from an underground bore, which is filtered using mesh filters and UV treatment. The F_1_ generation was produced using hormone‐induced mass spawning in December 2015. Induced spawning of F_0_ individuals was achieved subsequent to an intramuscular injection of human chorionic gonadotropin (hCG, Chorluon^®^) at a target dose of 600 IU/kg of bodyweight. Leading up to spawning, surviving parents (*n* = 19) were fed a specialized diet containing fresh fish and oil supplements. Following injection, two individuals became egg bound and died, most likely before spawning. Spawning occurred 48 h post‐injection of hCG. Fifty grams of eggs were collected each day from the tank outlet over three consecutive days and placed in 450 L hatchery tanks provided with a 12‐h light cycle, gentle aeration and water flow. At seven days post‐hatching, larvae were combined into a single 5000 L tank. The larvae were fed a combination of live rotifers and artemia. At 1 month old, the juveniles were then fed a combination of artemia, O.range (NRD) dry crumbs, and a wet diet consisting of minced fresh fish. At 6 months of age, all fish were transferred into a single 5000 L tank with natural lighting and fed a combination of dry commercial pellets (Skrettings Nutra RC 1.2 mm and 1.8 mm) until 1 year old, and then Ridley pellets (2 mm, 3 mm, 4 mm and 6 mm) and a wet diet (fish mince or portions of fresh fish). In November 2017, at 2.1 years old, 1100 F_1_ were randomly selected and transferred to a single 13,000 L tank. During this time, all fish were tagged by inserting a Passive Integrated Transponder (PIT) tag (GPT12, Biomark^®^) into the body cavity. To minimize the risk of overcrowding and stunting of growth, F_1_ was moved for a final time four months later (March 2018) to a single 50,000 L tank and were maintained under same ambient temperatures and photoperiod for the remainder of the experiment.

### 2.2 Tissue sampling, DNA extraction and library preparations

Thirteen surviving F_0_ were tagged and fin‐clipped in January 2017. Fin clips were placed directly into chilled 96% ethanol, heated to 80°C for 5 min within 1 h of collection, and then stored at −20°C until needed. Total DNA was extracted as described by Ashton, Hilario et al. (2019), Ashton, Ritchie et al. (2019) with the following modifications: proteinase K digestion time was increased to 1.5 h and the 80°C inactivation step was omitted; the RNA removal was performed after the salting‐out step; and the DNA was quantified by fluorescence using the Qubit High Sensitivity dsDNA kit (Thermo Fisher Scientific) used in accordance with the manufacturer's instructions. DNA quality was assessed by agarose gel electrophoresis (average fragment size ~40 kbp) and using spectrophotometry (absorbance ratios at 260/280 nm and 260/230 nm). Illumina shotgun fragment libraries with an insert size of at least 125 bp were generated for each of the 13 individuals and sequenced (paired‐end, 125 bp reads) over three lanes of the HiSeq 2500 platform at the Australian Genome Research Facility (AGRF). Samples of fin tissue from 1100 F_1_ individuals were collected during the first phenotyping round (November 2017) and stored as described above. Total genomic DNA was extracted by SlipStream Automation using the same protocol as for the F_0_. Genotyping for the F_1_ was carried out using a modified GBS approach (Elshire et al., 2011; Hilario, 2015). DNA integrity was checked by capillary electrophoresis (High Sensitivity genomic DNA kit), Fragment Analyzer (Advanced Analytical). One microgram of total genomic DNA was used for digestion with restriction enzymes. A double digestion was performed with *Pst* I and *Msp* I by incubation at 37°C for 3 h, the adaptor ligation step omitted drying out the DNA/adaptor mixture. The barcoded adaptors were associated with the *Pst* I cut sites and designed by Deena Bioinformatics. Adaptors were annealed according to Ko et al. (2003). A high‐fidelity enzyme was used for amplifications (AccuPrime Taq DNA polymerase High Fidelity, Life Technologies). Amplification, quality check and clean up were done separately before pooling samples. A total of 12 pools of 96 samples each were prepared and sent to AGRF for sequencing on a HiSeq 2500 platform (single‐end, 100 bp reads).

### 2.3 Genotyping data quality checking and processing

Sequencing data quality for both F_0_ and F_1_ generations were checked using FastQC v0.11.7 (Andrews, 2010). As the F_0_ and F_1_ sequence data were generated using different sequencing technologies, different filtering parameters were used.

#### 2.3.1 F_0_ filtering/pre‐processing

Raw reads from the F_0_ were trimmed using trimmomatic v0.36 (Bolger et al., 2014) (using the parameters HEADCROP: 9, TRAILING: 10, SLIDINGWINDOW: 5:20, MINLEN: 75). Read groups were added and bam files were sorted and indexed using Picard toolkit (Toolkit, 2015). The trevally reference genome developed by PFR (Ruigrok et al., 2021) was indexed using Burrows‐Wheeler Aligner (BWA) v0.7.17 (Li & Durbin, 2009). Reads were aligned to the reference using BWA‐mem and the variant calling was done using samtools v1.9 and BCFtools v1.9 (Li, 2011): the samples were combined using mpileup, and the call was run using the multiallelic caller option. A first round of filtering was then done using VCFtools v0.1.14 (Danecek et al., 2011). Briefly, indels were removed from the parental call, which was subsequently filtered for high missing rates per individuals (threshold set at 0% missingness) and minimum SNP quality and depth (Q > 10, DP > 9). A further filter was applied for missing data per SNPs (set at 0% missingness). Finally, SNPs were filtered for maximum depth (maxDP = average DP + 3 standard deviations = 445).

#### 2.3.2 F_1_ filtering/pre‐processing

The F_1_ samples were de‐multiplexed from the 12 sequencing libraries using the process_radtags module available in the STACKs v2.1 pipeline (Catchen et al., 2013), and the reads were trimmed using Fastq‐mcf in ea‐utils v1.1.2‐806 (minimum sequence length = 50, quality threshold causing base removal = 33) (Aronesty, 2013). Like for the parents, read groups were also added and the bam files were sorted, indexed and aligned to the reference using BWA‐mem. The variant calling was also done using samtools and BCFtools. Using VCFtools, indels and individuals with over 50% missing data were removed, the maximum depth was set at 8000 and SNPs with over 20% missing data were filtered.

#### 2.3.3 Recombining datasets

After indexing, a list of common SNPs between F_0_ and F_1_ was obtained using BCFtools isec and the two sets were merged using VCFtools vcf‐merge. Finally, the resulting F_0_ and F_1_ dataset was filtered to keep only SNPs in common using VCFtools −positions.

### 2.4 Pedigree reconstruction and F_0_ sex prediction

Sequoia v2.0.7 (Huisman, 2017) was used in the R statistical environment (version 3.2.3) (R Core Team, 2013) to iteratively reconstruct a maximum‐likelihood pedigree. To prepare the common SNP dataset for pedigree reconstruction, PLINK v1.9 (Purcell et al., 2007) was first used to test for and discard loci in linkage disequilibrium (LD) with the ‐‐indep function, evaluating 50 SNP windows, five SNPs at a time, with a variance inflation factor (VIF) cut‐off = 1.5 and a minor allele frequency (MAF) of 0.4 was set for the population. The initial parentage assignment was accomplished with this genotype file and a life history file, using the parameters MaxSibiter = 0, Err = 0.05, MaxMismatch = 10, MaxSibshipSize = 900 and Tassign = 0.5. This allowed to scan the pedigree for obvious errors, as well as for duplicates that were accidentally retained. To construct the full pedigree, the parameter data frame (= Specs) was then altered to use the initial parentage assignment as prior information and MaxSibIter = 3, MaxSibshipSize = 900, Err = 0.2, Tfilter = −2, Tassign = 0.5. All other parameters were kept as default. We assessed the accuracy of the reconstructed pedigree and the ability of the SNP data set to correctly identify familial relationships by checking for Mendelian errors using PLINK.

Broodstock sex prediction based on the molecular pedigree was confirmed a posteriori by collecting a gonadal biopsy from each of the 13 F_0_ individuals in December 2018. In brief, broodstock in the advanced stages of reproductive development were mass‐sedated in tank (25 ppm Aqui‐S; Aqui‐S New Zealand Ltd). A gonadal biopsy was taken by inserting a glass cannula (Natelson tube, 3 mm outside diameter) connected to a plastic tubing into the gonopore of the fish and applying gentle aspiration by syringe. A portion of biopsy sample was placed in Ringer's solution (180 mM NaCl; 4 mM KCl; 1.5 mM CaCl_2_; 1.2 mM MgSO_4_; 3 mM NaH_2_PO_4_; 12.5 mM NaHCO_3_ – pH 7.5), and from this, a wet mount slide was prepared and examined under a compound microscope for the presence of oocytes or sperm.

### 2.5 Inbreeding calculations

Using the same set of filtered SNPs used for the parentage assignment, inbreeding values for each individual found with both parents in the pedigree were calculated with a method‐of‐moments F coefficient (*F*_H_) using PLINK. This statistic is equal to Nei's *F*_IS_ statistic, but is calculated using a different formula:

$$F_{h}=\frac{O\left({\mathrm{Hom}}_{i}\right)‐E\left(\mathrm{Hom}\right)}{m‐E(\mathrm{Hom})}$$

where *O*(Hom*_i_*) is the observed number of homozygous loci for the *i*th individual, and *E*(Hom) is the Hardy–Weinberg expected mean number of homozygous genotypes across *m* loci (Kardos et al., 2015). The distribution of inbreeding values was then visualized using the ggplot2 library in R. A Welch two‐sample *t* test was used to compare mean inbreeding values between F_0_ and F_1_. To compare mean inbreeding values between families, a linear mixed model was fitted with family as a random effect to run an ANOVA. A post hoc Tukey correction for multiple comparisons was used to find which family values were significantly different from the others.

### 2.6 Phenotyping, trait estimations and phenotypic correlations

In total, eight sets of external images were taken for each F_1_ individual, corresponding to a measurement roughly every four months during a period of two years. Images were taken on a custom‐built imaging rig using fitted Panasonic Lumix DMC‐GH4 cameras. Images were analysed using PFR’s Morphometric Sotfware™ (https://www.plantandfood.co.nz/page/morphometric‐software‐home/). The software extracts the outline of each individual fish from images, locates the XY coordinates of morphometric features on the outline (e.g. upper lip and narrowest cross section of the tail) and then uses those coordinates to make measurements. The measurements were converted from pixels to mm using the length of rulers also present in the images.

**FIGURE 1:**
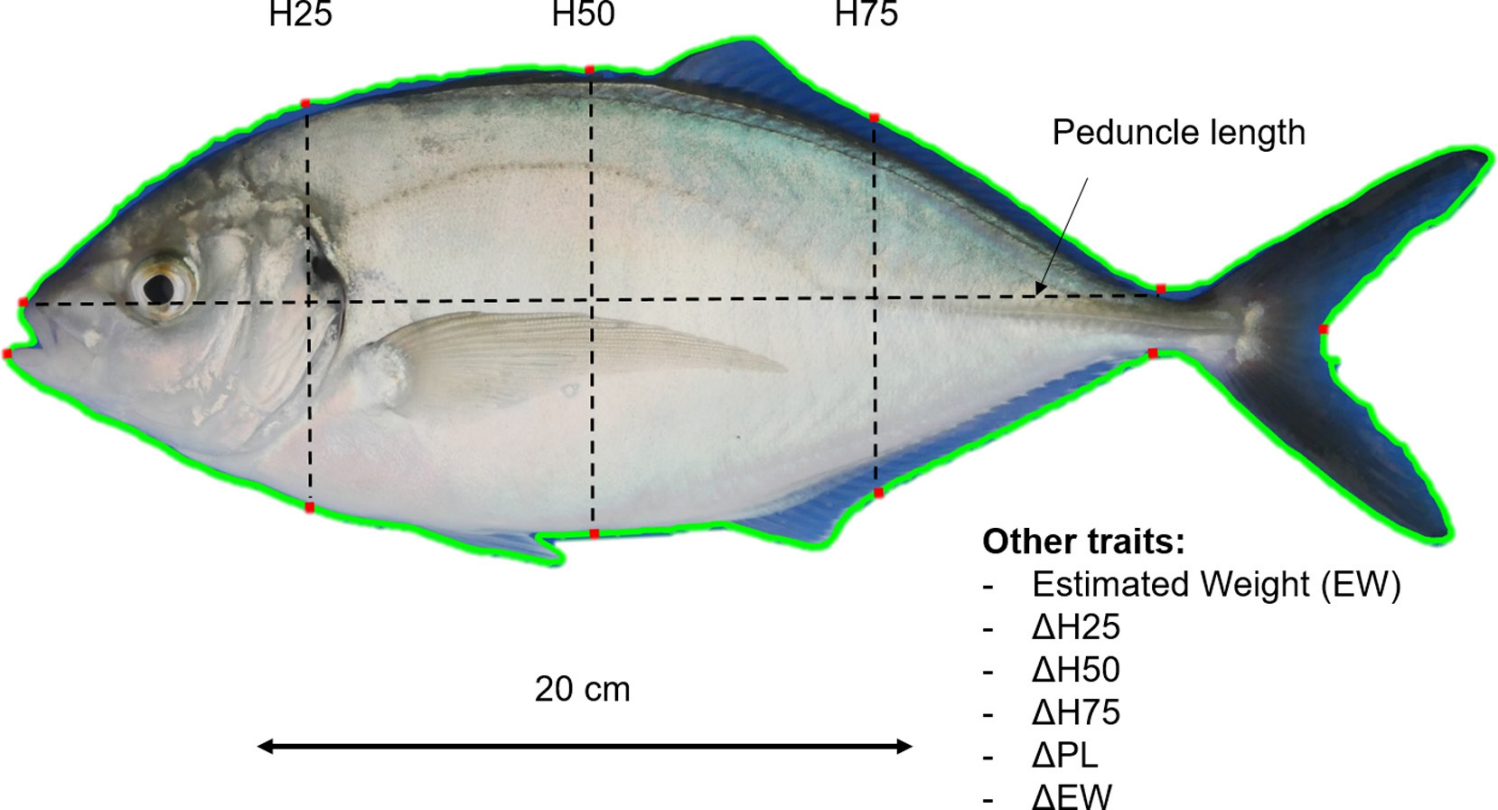
Morphometric traits (PL: peduncle length, H25: height at 25% of PL, H50: height at 50% of PL, H75: height at 75% of PL, EW: estimated weight, ΔH25: net gain in H25, ΔH50: net gain in H50, ΔH75: net gain in H75, ΔPL: net gain in PL, ΔEW: net gain in EW), measured in the New Zealand silver trevally *Pseudocaranx georgianus*. Peduncle length, and the three height measurements were obtained automatically using a custom image analysis script. Height measurements were measured relative to length. Weight was estimated using a Bayesian hierarchical approach (Froese et al., 2014). Net traits were calculated using the first measurements as initial point

Four growth traits were directly extracted from the pictures for each individual fish. Peduncle length (PL) was measured by locating the upper lip and narrowest cross section of the tail and then measuring the distance between these points. Height was measured at three positions along the fish – 25%, 50% and 75% of the way from the upper lip to the narrowest cross section of the tail (H25, H50 and H75, respectively, Figure 1). To do this, the software first located a starting position along the peduncle length and then measured the distance between the top and bottom edge of the fish at a 90° angle to the peduncle length.

In addition, six other traits were estimated: weight (EW), and net gains in height at – 25%, 50% and 75% of PL (ΔH25, ΔH50 and ΔH75, respectively), in peduncle length (ΔPL) and in weight (ΔEW). The weight estimations were done following Froese et al. (2014), using a Bayesian hierarchical approach. Briefly, a set of manually recorded measurements from a subgroup of 143 F_1_ trevally was used to generate the parameters used to predict weight (*W*) from length (*L*) using the length–weight relationship:

$$W=aL^{b}$$

where parameter *b* indicates growth in body proportions as the slope of a regression over log‐transformed weight‐at‐length data, and *a*, the parameter describing body shape, as the intercept of a regression line over log‐transformed weight‐at‐length data. The accuracy of the prediction model was estimated by calculating its *R*‐square score. The net gain in each trait for each time point was calculated as the difference between the initial measurement in November 2017 and the measure of that month.

The phenotypic correlations between individual traits were measured using Pearson's correlation matrix, which was constructed using all phenotypic measurements in Python v2.7, using the Numpy library (McKinney, 2010).

### 2.7 Trait heritability and genetic correlations

Variance and covariance components were estimated using linear mixed animal models and restricted maximum likelihood methods with ASREML version 4.0 (Gilmour et al., 2015) in R. Narrow‐sense heritability of each trait was estimated using a univariate analysis, modelled as follow:

$$y_{i}=\mu +a_{i}+e_{i}$$

where μ is the population mean, $a_{i}$ is the breeding value and $e_{i}$ is a residual term (Galwey, 2014). The heritability models were run separately for each time measure, with the target trait predicted using a fixed intercept effect. The genetic covariances were estimated in a series of bivariate analyses. A bivariate model was fitted for the trait combinations to estimate genetic correlations and their standard errors, using the equation:

y=Xβ+Zu+e

where ***X*** and ***Z*** are matrices and ***y***, ***u*** and ***e*** are vectors (Thompson et al., 1995).

### 2.8 Ethics

All research carried out in this study was approved by the animal ethics committee of Victoria University of Wellington, application number 25976. All data used in this study including the genome assembly, WGS and GBS sequencing libraries, phenotype data and supplemental material will be deposited in an open data repository, which will be accessible via www.genomics‐aotearoa.org.nz/data.

## 3 RESULTS

### 3.1 Filtering allowed to increase quality of calls tenfold in offspring

A total of 1.23 billion DNA sequence reads were generated for the F_0_, resulting in 13x coverage for each of the 13 individuals; a total of 3.05 billion reads were produced for all 12 pooled libraries of F_1_, with approximately 3 million sequence reads for each individual library, resulting in 0.42x genome coverage per individual fish (Table S1). Quality was high across the full length of the reads for all F_1_ plates, except towards the end of plate 5, where quality was slightly reduced (Figure S1). Twenty‐three F_1_ sample extractions did not yield enough DNA for sequencing. A further 29 out of 1077 offspring libraries failed to be sequenced, probably due to low quality DNA.

The initial variant calling yielded 20.8 and 2.1 million markers for the F_0_ and F_1_, respectively (Figure S2A–D). After removing indels, 17.8 million sites were kept for the F_0_ and 1.8 million for the F_1_. Forty‐seven offspring were subsequently removed based on missing data (Figure S2E,F). The average SNP read depths were 181.99 ± 86.57 (min: 16; max: 4261) for the F_0_ and 5126.33 ± 1696.81 (min: 1759; max: 8000) for the F_1_. After filtering for quality and depth, 17.7 and 1.1 million SNPs were kept for the F_0_ and F_1_, respectively. The missing rate per SNP filtering resulted in 17.1 million and 214,700 sites for the parents and the offspring, respectively (Figure S2G,H). The last filter for maximum depth in the parental call retained 16.9 million SNPs. These filtering steps resulted in a total of 171,923 SNPs shared between the F_0_ and F_1_ generations. Filtering enhanced largely the F_1_ dataset, going from 68% missing data to 0.06%. The parental dataset went from 0.01% to 0% (Table S1).

### 3.2 Reconstruction of the pedigree showed skewed parental contributions

**FIGURE 2:**
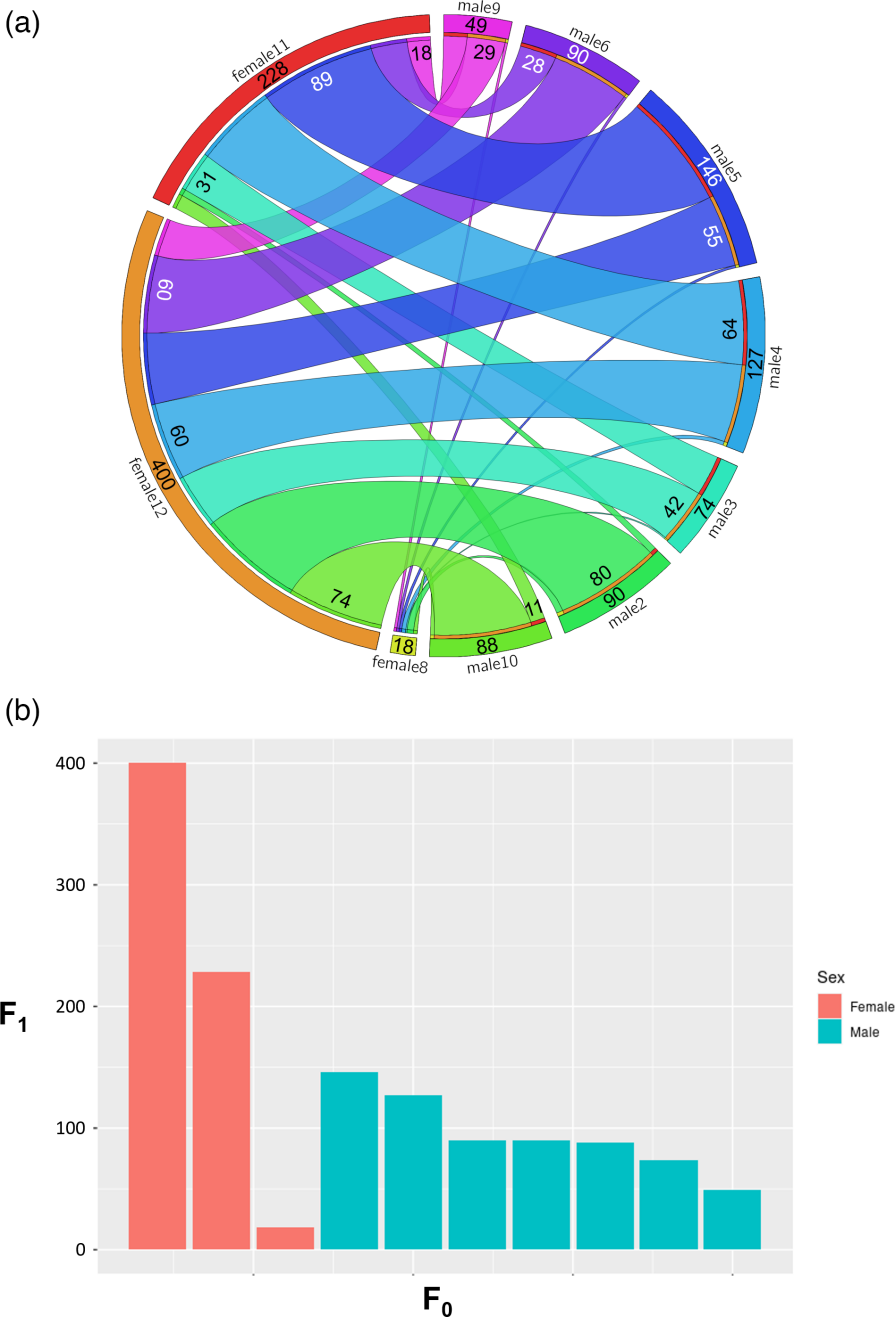
Molecular pedigree of the trevally population. (a) Circos representation of the pedigree structure of the population of trevally. 21 families were identified. Numbers in the outer in the outer ring indicate the total number of offspring per parent. Numbers in the ribbons indicate the number of offspring in the family. (b) Number of offspring produced by each parent in the F_0_ generation divided into females (red) and males (blue)

A subset of 1525 SNPs was used for the parentage assignment. Pedigree reconstruction allowed to determine sex for all F_0_ individuals, which was further confirmed by biopsy. Visualization of the pedigree showed that out of 13 sequenced F_0_, 10 individuals participated in the spawning (three females and seven males), generating 21 families (Figure 2a). Both parents were identified for 63% (664) of the individuals in the F_1_ population. The remaining F_1_ had either one (31.4%) or both parents (3.6%) not genotyped. Although the mating ratios were equal among all females (1:7) and males (1:3), the contributions were skewed among both sexes, particularly so in females, with one of them contributing to up to 60.2% of the total F_1_ population (Figure 2b).

### 3.3 Unbalanced inbreeding levels detected within families

**FIGURE 3:**
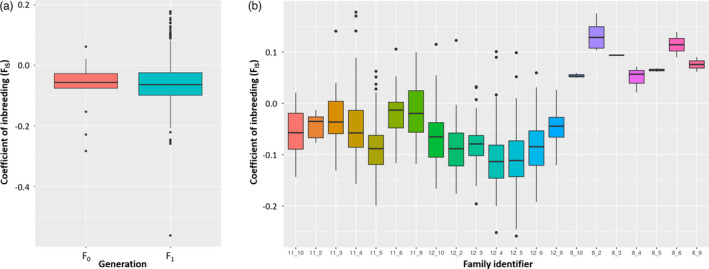
Inbreeding scores for trevally individuals based on (a) generation, and (b) family. Visualized are the 1st, 2nd (median) and 3rd quartile and whiskers extending 1.5 times the interquartile range from the median (95% confidence interval). The coefficient of inbreeding used is Nei's *F*_IS_ and significant differences between groups are shown in Table S2. No significant differences (*p*‐value > 0.01) were found between the F_0_ and F_1_ generations. Significant differences (*p*‐value < 0.01) were found between different families

The inbreeding F statistic (*F*_H_) was calculated for 13 F_0_ and 664 F_1_ individuals with both parents known. The values ranged from a minimum of −0.28 to a maximum of 0.06 with a median of −0.06 for the F_0_ and from −0.56 to 0.18 with a median of −0.06 for the F_1_. Variation in the inbreeding values did not significantly differ between the wild‐caught F_0_ and the F_1_ generation (−0.08 to −0.06, respectively, *p*‐value = 2.65^−05^) (Figure 3a). Most of per family average inbreeding values ranged from −0.02 to −0.11 except for families having female 8 as mother, which had higher average *F*_H_ values, ranging from 0.06 to 0.14 (Figure 3b). The mean inbreeding values for fam11_5, 12_2, 12_4 and 12_5 were significantly lower (ρ < 0.01) than those of other families (Table S2). Families with female 8 as a mother had a sample size too low (*n* = 1 to *n* = 5) to conclude if the means were statistically significantly different from those of the other groups.

### 3.4 Growth pattern in trevally is seasonally influenced and is under family effects

For 2 years, phenotyping of the F_1_ trevally cohort occurred roughly every 4 months, from the age of 2.1 to 4.1 years old. Ten growth traits that could either be directly extracted from the images (H25, H50, H75 and PL), indirectly measured as in the case of weight, or calculated a posteriori, as in the case of the net gains traits, were recorded across eight time points. The *a* and *b* parameters used in the Bayesian hierarchical approach (Froese et al., 2014) were 0.02 and 2.99, respectively. They predicted weight with an accuracy of *R* ^2^ = 0.92. The number of measurements per time point differed as a result of natural mortality and access to the individuals within the tanks. Between the first and second time points, the most significant drop was observed from 1093 to 748 individuals, because of a high number of PIT tags being rejected from the gut cavity. The increase in sample size in the last measurement is explained by the fact that all individuals could be retrieved from the tank.

**FIGURE 4:**
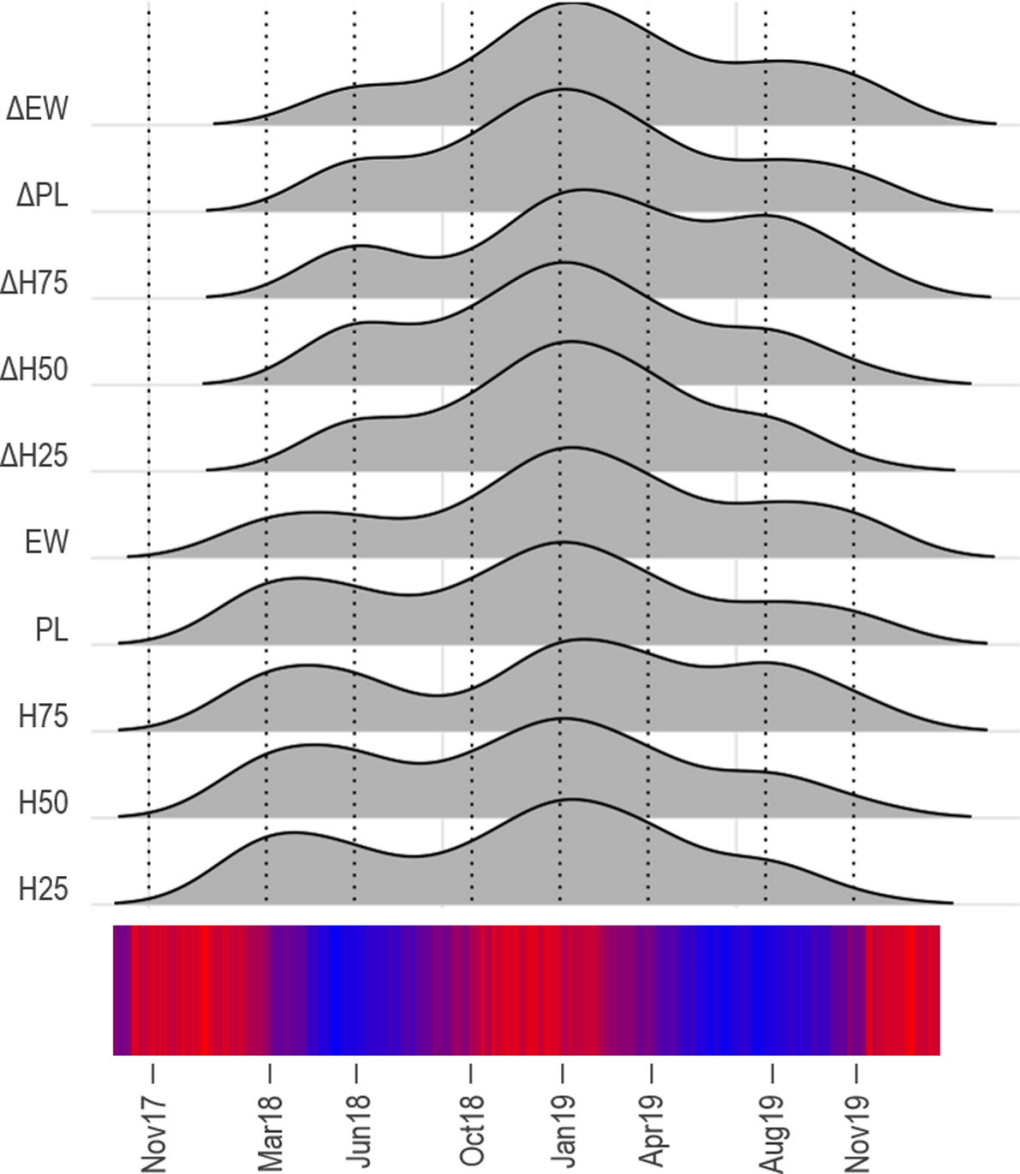
Influence of seasonal temperature on the growth rate of trevally over two years for peduncle length (PL), height at 25% of PL (H25), height at 50% of PL (H50), height at 75% of PL (H75), estimated weight (EW), net gain in H25 (ΔH25), net gain in H50 (ΔH50), net gain in H75 (ΔH75), net gain in PL (ΔPL) and net gain in EW (ΔEW). The area under the curve represents the relative percentage of gain measured in November 2017 (Nov17), March 2018 (Mar18), June 2018 (Jun18), October (Oct18) 2018, January 2019 (Jan19), April 2019 (Apr19), August 2019 (Aug19) and November 2019 (Nov19). The bottom band indicates the ambient temperature recorded in the tank

High variation was observed in all traits (Figure S3). Between the first and the last measurements, standard deviation less than doubled for all traits but increased fourfold for EW and ΔEW (Table S3). Coefficients of variation (CV) of height, length and weight traits remained constant throughout the experiment showing that variability in relation to the mean of the population was stable. The CV of net gain traits were inflated in the first measure (March 2018) and decreased over time (November 2019) (Figure S4). Growth increase was not linear throughout the duration of the experiment. In all traits recorded, a seasonal pattern was detected (Figure 4). Higher gains in all traits were concomitant with warmer ambient water temperatures. During summer (December to February), growth increased by an average of 27.3% across all traits (min: 22.2%, max: 31.1%), whereas during winter, growth only increased 7% on average (min: 2.3%, max: 12.3%).

When subdividing the F_1_ by families, full‐sibs with higher initial measures for traits H25, H50, H75, PL and EW remained higher throughout the experiment (Figure S5A–E). For instance, families 11_10 and 11_3 had the highest average measures of H25 in November 2017 with 61.28 mm and 64.00 mm, respectively, and 106.88 mm and 110.83 mm, respectively, in November 2019. However, gains in net growth traits (ΔH25, ΔH50, ΔH75, ΔPL and ΔEW) did not follow the same trend: compared with family 11_10 scoring the highest values for the measured traits across time, family 11_5 had a higher final net gain in all traits but ΔEW (Figure S5F–J).

### 3.5 Genetic correlations, phenotypic correlation and trait heritability

The estimates of heritability, variances, covariances and phenotypic correlations between traits are reported in Table S4.

**FIGURE 5:**
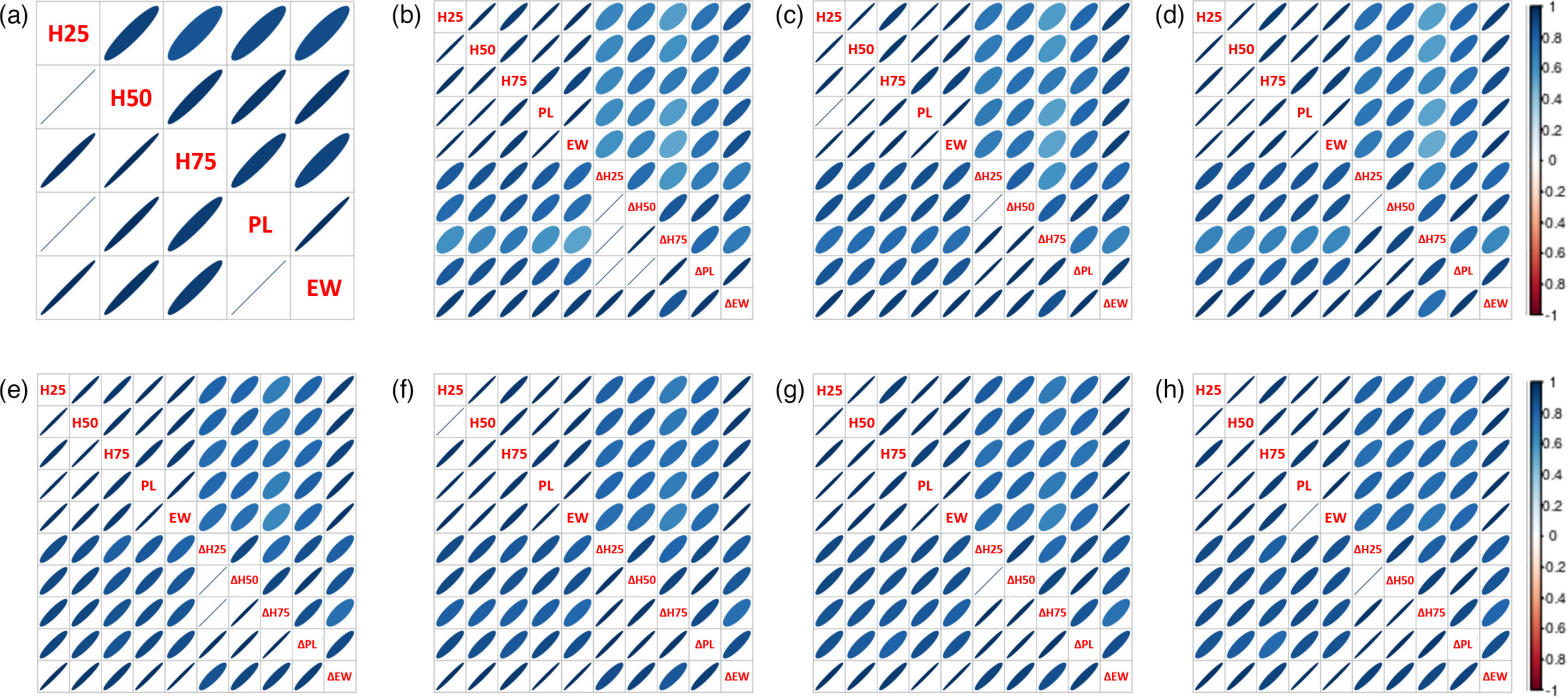
Heat maps of Pearson's phenotypic correlations (above the diagonal) and genetic correlations (bellow the diagonal) in (a) November 2017, (b) March 2018, (c) June 2018, (d) October 2018, (e) January 2019, (f) April 2019, (g) August 2019 and (h) November 2019, between height at 25% (H25), height at 50% (H50), height at 75% (H75) of the peduncle length (PL), estimated weight (EW) and net gain in height 25% (ΔH25), height 50% (ΔH50), height 75% (ΔH75), peduncle length (ΔPL) and estimated weight (ΔEW). Positive correlations are displayed in blue and negative correlations in red colour. Colour intensity and the size of the circle are proportional to the correlation coefficients

Based on Pearson's correlation coefficients, strong phenotypic correlations were observed between all height, length and weight traits throughout the experiment (0.85 ± 0.02 to 1.00 ± 0.00) and moderate to strong correlations were found for the net growth traits (and 0.50 ± 0.00 to 0.98 ± 0.01) (Figure 5).

Narrow‐sense heritability was estimated for all phenotypic traits. Heritability estimates remained consistent throughout the experiment. The heritability range was moderate to high (0.67 ± 0.05 to 0.76 ± 0.06) for the measured traits (H25, H50, H75, PL and EW) and moderate (ranging from 0.28 ± 0.07 to 0.68 ± 0.07) for the net gain traits (ΔH25, ΔH50, ΔH75, ΔPL and ΔEW).

Strong positive genetic correlations were found between all measured height, length and weight traits throughout the experiment (close to unity, 0.94 ± 0.00 to 1.00 ± 0.00) and between all net gain traits (0.84 ± 0.07 to 1.00 ± 0.03). Although calculations of weights were made using length measurements, such high genetic correlations indicate that the alleles associated have a pleiotropic effect and influence multiple traits simultaneously, Correlation between measurements of height, length, weight and net gain traits were genetically moderate to strong (0.59 ± 0.10 to 0.99 ± 0.00).

The genetic variance of each trait increased over time, especially for length and weight traits (PL: 149.87 in November 17 to 524.09 in November 19; EW: 458.59 to 9178.11 in November 19; ΔPL: 98.70 in March 18 to 388.12 in November 19; ΔEW: 477.98 in March 18 to 7194.48 in November 18), showing that variation between individuals increases as fishes get bigger.

## 4 DISCUSSION

Here, we applied, for the first time, a genomics‐informed approach to study a captive trevally population in New Zealand. Data from different genotyping methods were pooled to reconstruct a two‐generation pedigree of 13 F_0_ and 1100 F_1_ and to investigate the inbreeding levels in each generation and within families. Phenotyping data collected over 2 years were added to assess the quantitative genetic architecture of 10 growth traits. The results of this study will support breeding efforts in trevally, as well as inform more generally genomic work on other teleost species.

Marker‐based pedigree reconstruction enabled to determine the sex of the broodstock and showed that most individuals contributed to F_1_ offspring. However, reproductive contributions were skewed, particularly among females. Skewed contributions have been reported from a wide range of captive fish populations, including closely related species such as the yellowtail kingfish (Dettleff et al., 2020) and geographically co‐occurring species such as the Australasian snapper (*Chrysophrys auratus*) (Ashton, Hilario et al., 2019; Ashton, Ritchie et al., 2019). Because this study was carried out on F_1_ obtained via hormone‐induced spawning, different explanations could account for this biased distribution. First, physiological states of hCG‐injected individuals (e.g. different stages of the reproductive cycle) may have influenced their ability to spawn, or limited gamete availability. Second, different survival rates of progeny could have skewed family representations (note: DNA sampling of the F_1_ was conducted at 2.1 years of age). Third, courtship and mating behaviour might have been at play. This has been documented in yellowtail kingfish (Dettleff et al., 2020; Moran et al., 2007) where only one female and one male mated at any given time. Any of these factors, or a combination of them, could explain the skewed parental representation.

The initial level of genetic variation entering a breeding programme is set by the amount of heterozygosity of the founding population. It is crucial to monitor its subsequent loss in the next generations, as this will help to avoid inbreeding depression and a diversity bottleneck for a breeding programme. In our study, the wild F_0_ parents represented the baseline for inbreeding statistics. No statistical differences were observed between the two generations, which were both slightly outbred (−0.08 and −0.06 on average for F_0_ and F_1_, respectively). Although the average inbreeding rates did not vary between generations, some slight differences could be observed between families in the F_1_ generation. Values were similar to the ones found in other wild‐caught marine species such as orange clown fish (*Amphiprion percula*) (0.018) (Salles et al., 2016), Australasian snapper (0.055) (Ashton, Hilario et al., 2019; Ashton, Ritchie et al., 2019) or pacu fish (*Piaractus mesopotamicus*) (0.054–0.247) (del Pazo et al., 2021). Negative inbreeding coefficients can be the result of excess of observed heterozygotes, which, in our study, can be explained as an effect of the genetic drift caused by the sampling of our bloodstock from the wild population. We may have calculated low *F* values even in families whose parents had high kinship coefficients and therefore were expected to be highly inbred. Such *F* values will then quickly increase in later generations.

During our 2‐year study, we recorded 10 growth traits across eight time points to describe the growth patterns of trevally. All fish were maintained under the same rearing conditions throughout the duration of the experiment. Despite this, some external parameters, such as food availability, might have varied slightly for some individuals because of the potential for hierarchical social behaviour in confined pools, but this is expected to have contributed to only minimal variation. A certain level of trait variability is evident in the data because not all individual fish could be extracted and sampled at each sampling point for logistical reason, meaning that for some data points, some very large or small fish were not included (though at each sampling point around 80% of the fish could be measured). We found that families which had initially higher values in growth traits remained larger throughout the experiment. However, some smaller families showed higher net gains compared with the larger families, demonstrating that the bigger fish were not necessarily the fastest growing proportionally and could also indicate some degree of compensatory growth. Finally, growth showed a pattern of being strongly influenced by seasonal temperatures. An increase in growth rate was observed during the warmer months of the year (*T*°~ 21°C) compared with the colder months (*T*°~ 11°C). This can be explained by an increase in metabolism during summer as water temperature rises and day length increases (Pauly, 1980). Similar results have been found in other species such as in chinook/king salmon where the optimal growing temperature is 19.0°C (Perry et al., 2015), or in yellowtail kingfish, where optimal rearing temperatures were found to be around 26.5°C (Abbink et al., 2012), and Australasian snapper, which show increased growth rates at 21.0°C compared to 13.0°C (Wellenreuther et al., 2019). However, too high temperatures can also have a negative effect on growth rates once it exceeds a tolerance threshold, as shown in varied species of coral reef fishes (Munday et al., 2008).

Genomic‐based pedigree allowed the estimation of narrow‐sense heritability for measured growth traits, which were consistent over time and higher than those reported in other studies; for example, heritability estimates for growth traits (weight or length) ranged from 0.26 (Whatmore et al., 2013) to 0.42 (Premachandra et al., 2017) in yellowtail kingfish; 0.3–0.34 in Asian seabass (*Lates calcarifer*) (Ye et al., 2017); 0.09–0.30 in Australasian snapper (Ashton, Hilario et al., 2019; Ashton, Ritchie et al., 2019); and 0.42–0.72 in Atlantic salmon (*Salmo salar*) (Thorland et al., 2020). These estimates are population specific and can be inflated if traits are also influenced by non‐additive genetic effects (such as epistasis and dominance) (Ashton et al., 2017; Visscher et al., 2008; Wray & Visscher, 2008). It is also to be noted that estimates are often biased upwards due to difficulties to separate environmental and non‐additive genetic effects common to full‐sibs from additive genetic effects when full‐sib families are reared in separate tanks until tagging (Kause et al., 2005). In this study, the high estimates observed in directly measured traits (H25, H50, H75, PL and EW) could be explained by the holding conditions of the fish. Although all fish were kept in the same conditions, early rearing effects (e.g. smaller tank until March 2018) might have been confounded with non‐additive genetic variation. Heritability estimates of the net growth traits were more comparable with values previously reported for teleost species mentioned above (0.27–0.68). It is likely that correcting for the growth period that happened in a restricting environment, from hatch to November 2017, helped remove some of the early rearing effects influencing the heritability estimates on the main phenotypes. Individuals were substantially smaller in November 2017, their environment—a 13,000 L tank where density was higher, oxygen levels likely lower, and competition might have occurred for space and food—could have had more of an impact on growth during juvenile stages (where there is less variance) compared with ~6 months later when growth had increased. Thus, the differences in growth observed in November 2017 could more likely be due to limiting environmental resources stunting their growth. From March 2018 onwards, the environment was less likely to be a limiting factor and the genetic component better explained the observed differences.

### 4.1 Future directions and management implications

This study represents the first in‐depth genetic investigation of reproductive success and growth rates for a captive trevally population in New Zealand. Genome‐wide marker sets combined with reference genomes will be useful for a wide range of future applications. Investigating the basic genetic structure of a founding population yields fundamental insights into the biology of a species and is of primary importance when establishing a long‐term breeding programme. Furthermore, understanding underlying genetic mechanisms of growth can support informed decisions about how to selectively breed species to fast track gains, while at the same time ensuring the long‐term viability of the breeding programme as a whole (e.g. avoiding inbreeding).

In trevally, like in other teleost, seasonal effects have a significant influence on the realized growth rate. This finding has important implications for the selection of aquaculture locations and monitoring of optimal rearing temperatures; holding fish in a warmer environment could increase the growth rate and reduce the time taken to reach harvesting size. Our study showed that families exhibiting larger measurements initially, remained the largest individuals in the population throughout the duration of the experiment. Early removal of small individuals can thus be a useful hatchery‐management method to maximize the realized growth potential overall. However, if there is no accompanying genetic management plan, it can result in a genetic bottleneck as often only a few families are selected, which increases the risk of inbreeding depression over several generations (Kincaid, 1983). The implementation of genomic information will enable selection decisions to be made on earlier measurements (e.g. March 2018 measure) while confidently preserving genetic diversity in the programme. Early rearing effects are common in land‐based facilities, where populations are kept in separate or small tanks, and this can influence the estimates of heritability of commercially important traits. We found that the inclusion of the net growth for each trait can improve the estimate of heritability, particularly if environmental differences had an impact on early performance. Focusing selection based on the net gain traits could help reduce the impact of environmental effect on the heritability estimates that would otherwise be difficult to separate out.

The results of this research indicate that trevally have skewed parental contributions, which may be a consequence of uncontrolled tank‐based spawning or subsequent family‐specific mortality. A cohort mating strategy could be used to increase the number of parents contributing to even out the mating differences. Tank‐based spawning of broodstock selected based on genotype data could help select less closely related individuals in the next generation. This approach, also called walk‐back selection, can be mixed with additional steps that reduce skewed contribution, such as holding the broodstock in multiple tanks and standardizing the volumes of fertilized eggs from each tank. However, these techniques introduce environmental variation, which would need to be accounted for in downstream analyses. If walk‐back selection were to be implemented, further research would also be needed to determine its effectiveness in controlling inbreeding over multiple generations.

The high heritability estimates found in this study highlight that there is potential for making strong genetic improvements via selective breeding. Phenotypic and genotypic correlations between traits were all positive and moderate to strong. A breeding objective for increased fish length is thus expected to produce a positive response on the other traits measured in this study, provided that they have the same allometric relationship later in life. Future work on this species could focus on a single trait such as length, as it can be easily measured using high‐throughput phenotyping methods and has been shown to be highly genetically correlated with other commercially relevant traits. Indeed, the speed of phenotyping will become increasingly more important as the range of locations for image‐based phenotyping grows wider. Using correlated traits like length and body shape from images to move into high frequency underwater environmentally linked measurements will enable more complete Genotype × Environment × Phenotype studies rather than single point Genotype × Phenotype studies in the future. Taken together, these results suggest that trevally is a suitable future candidate for enhanced growth.

## CONFLICT OF INTEREST

The authors declare no conflict of interest.

## Supporting information

Supplementary Material

## Data Availability

All research carried out in this study was approved by the animal ethics committee of Victoria University of Wellington, application number 25976. All data used in this study including the genome assembly, WGS and GBS sequencing libraries, phenotype data and supplemental material will be deposited in an open data repository, which will be accessible via www.genomics‐aotearoa.org.nz/data.
