# Supplementary material for: Evaluating new species for aquaculture: A genomic dissection of growth in the New Zealand silver trevally (Pseudocaranx georgianus)
Source: Evol Appl. 2021 Jul 30;15(4):591–602. doi: 10.1111/eva.13281 (PMC9046765; doi:10.1111/eva.13281)
Supplement: Supplementary file 1 — Supplementary Material [file EVA-15-591-s001.docx]

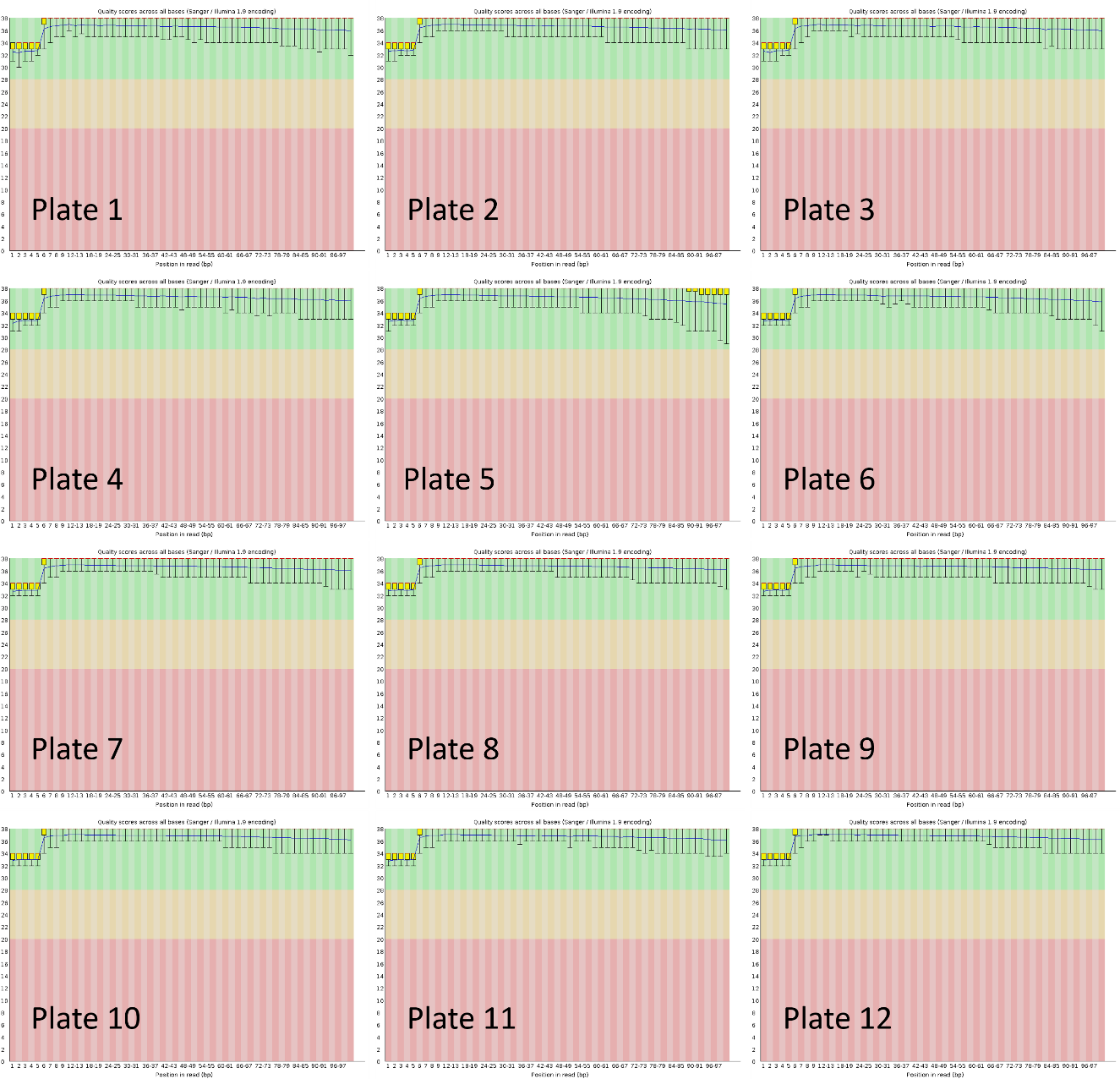


Figure S1: FastQC quality scores across the 100 bp reads for each of the twelve pooled sequencing plates of offspring.


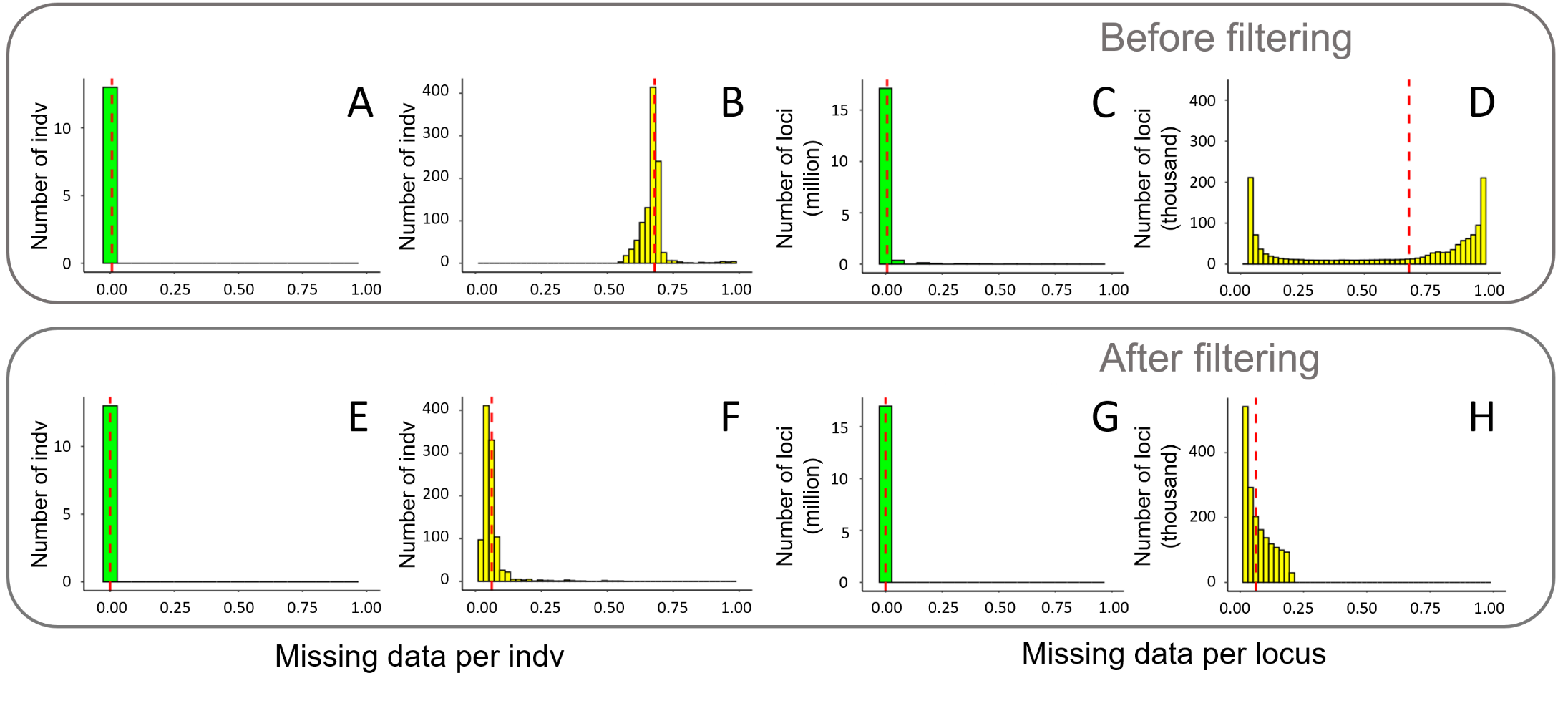


Figure S2: Filtering effects on the whole-genome resequenced F_0_ (green) and Genotyped-by-Sequencing F_1_ (yellow) data sets. Missing data per individual (indv) are plotted before and after filtering for the F_0_ (1 and 5) and the F_1_ (2 and 6) respectively; missing data per locus are plotted for unfiltered and filtered data sets for the F_0_ (3 and 7) and the F_1_ (4 and 8). The red dashed lines indicate the mean proportion of missing data.


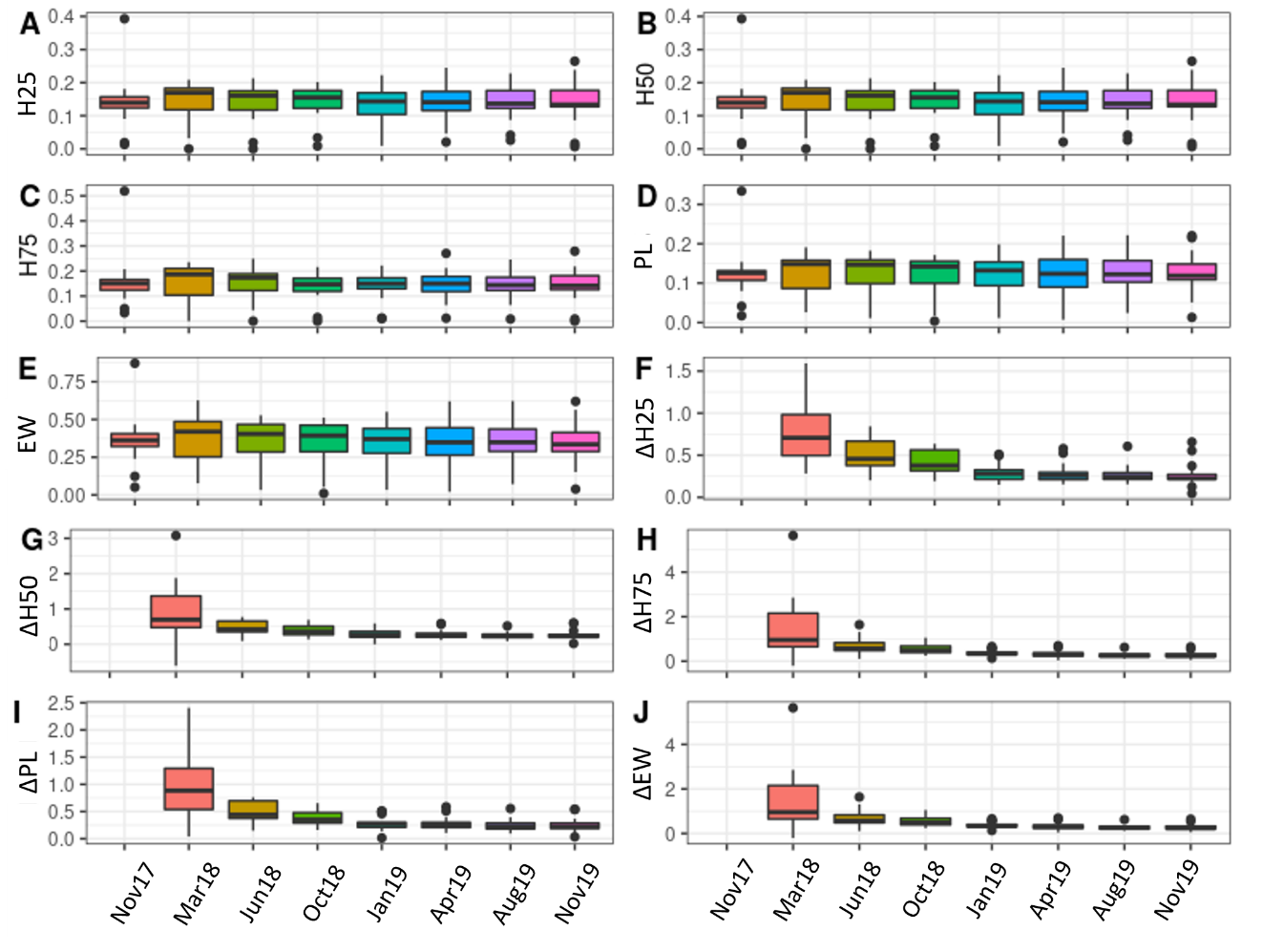


Figure S3: Distributions of the mean values for height at 25% of the peduncle length (H25), B) height at 50% of the peduncle length (H50), C) height at 75% of the peduncle length (H75), D) peduncle length (PL), E) estimated weight (EW), F) net gain in H25 (ΔH25), G) net gain in H50 (ΔH50), H) net gain in H75 (ΔH75), I) net gain in PL (ΔPL), J) net gain in EW (ΔEW) of captive silver trevally. The 25th, 50th and 75th percentiles are represented by horizontal lines and the 5th and 95th percentiles are represented by error bars. Dots represent outliers.


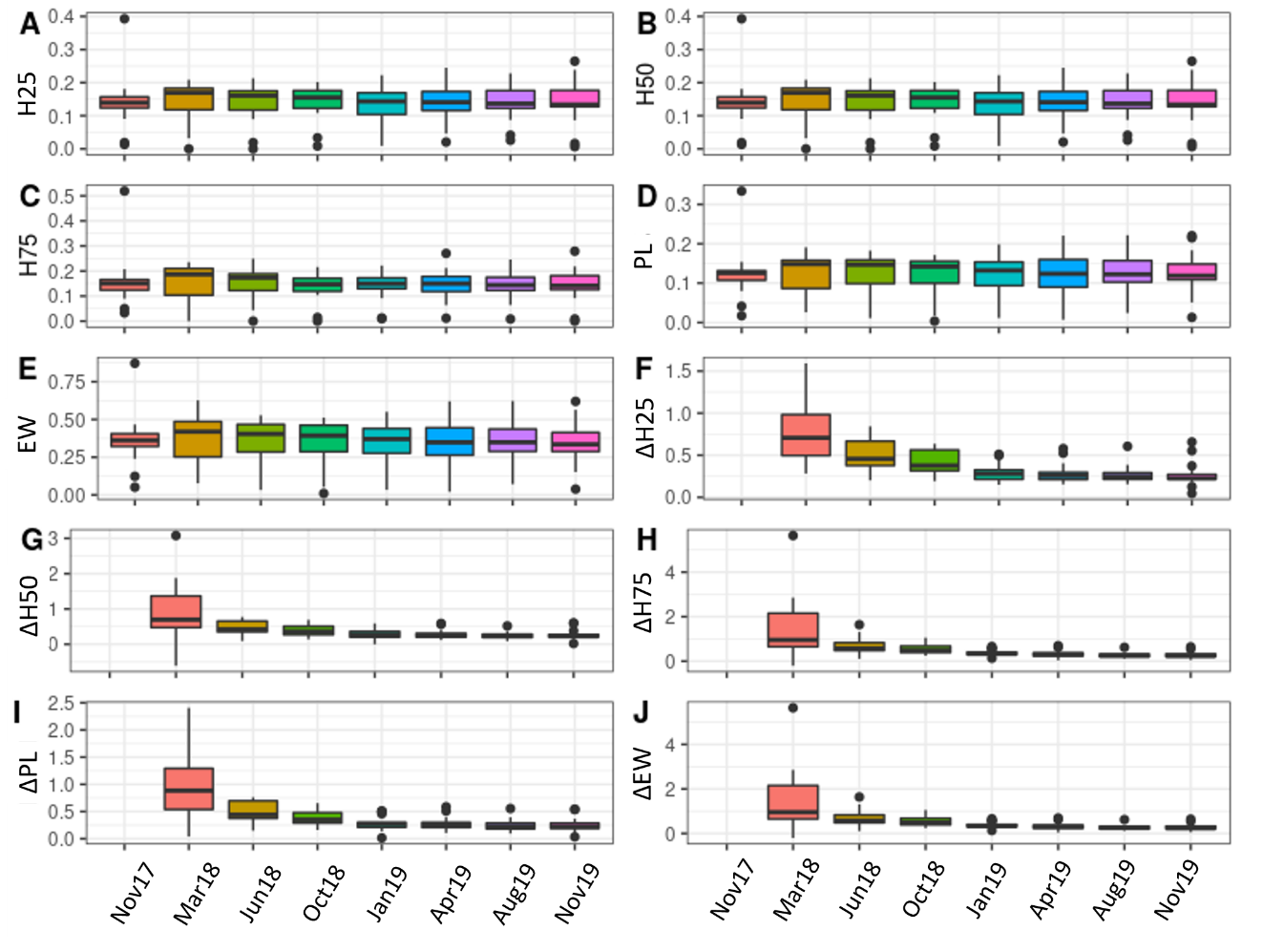


Figure S4: Distribution of the coefficient of variation of height at 25% of the peduncle length (H25), B) height at 50% of the peduncle length (H50), C) height at 75% of the peduncle length (H75), D) peduncle length (PL), E) estimated weight (EW), F) net gain in H25 (ΔH25), G) net gain in H50 (ΔH50), H) net gain in H75 (ΔH75), I) net gain in PL (ΔPL), J) net gain in EW (ΔEW) of captive silver trevally. The 25th, 50th and 75th percentiles are represented by horizontal lines and the 5th and 95th percentiles are represented by error bars. Dots represent outliers.


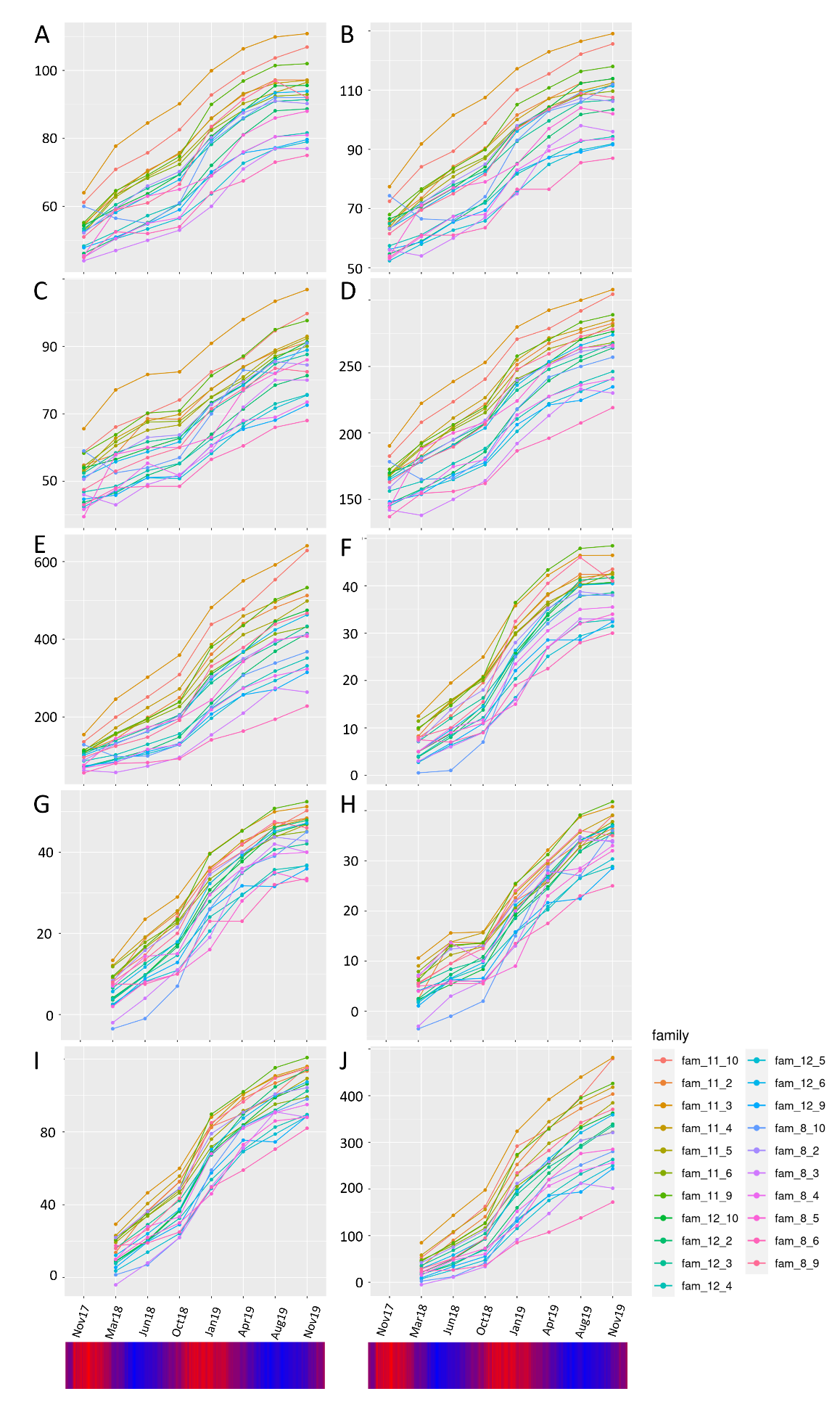


Figure S5: Growth rates per family for 10 morphometric traits A) height at 25% of the peduncle length (H25), B) height at 50% of the peduncle length (H50), C) height at 75% of the peduncle length (H75), D) peduncle length (PL), E) estimated weight (EW), F) net gain in H25 (ΔH25), G) net gain in H50 (ΔH50), H) net gain in H75 (ΔH75), I) net gain in PL (ΔPL), J) net gain in EW (ΔEW)

Table S1: Sequencing metrics and filtering effects on the whole-genome resequenced F_0_ and Genotyped-by-Sequencing F_1_ datasets.

|  | F_0_ | F_1_ |
| --- | --- | --- |
| Read length | 125 | 100 |
| Sequence reads (billion) | 1.23 | 3.05 |
| Coverage | 13 | 0.42 |
| Failed samples | 0 | 29 |
| Raw calling yields (millions of SNPs) | 20.8 | 2.1 |
| Indel filtering (millions of SNPs) | 17.8 | 1.8 |
| Removed individuals with missing data | 0 | 47 |
| Average read depth | 181.99 | 5,126.33 |
| Depth filtering (millions of SNPs) | 17.7 | 1.1 |
| Filter missing SNPs (millions of SNPs) | 17.1 | 0.2 |
| Maximum depth filter (millions of SNPs) | 16.9 | 0.2 |
| Total shared SNPs | 171,923 | |

Table S2: Comparison of the differences of the mean F statistic (F_IS_) between the F1 families. Upper triangle shows the *p*-values adjusted for multiple comparison using a post hoc Tuckey correction. The diagonal shows the means inbreeding value for each family. The bottom line shows the number (n) of offspring in the family.

Table S3: Count, mean, standard deviation (Stdv), and coefficient of variation (CV) for each continuous trait for in November 2017 (Nov17), March 2018 (Mar18), June 2018 (Jun18), October 2018 (Oct18), January 2019 (Jan19), April 2019 (Apr19), August 2019 (Aug19) and November 2019 (Nov19).

| Time measure | Trait | Samples | Mean | Stdv | CV |
| --- | --- | --- | --- | --- | --- |
| 17-Nov | H25 (mm) | 1093 | 49.71 | 9.03 | 0.18 |
|  | H50 (mm) | 1093 | 59.45 | 11.02 | 0.19 |
|  | H75 (mm) | 1093 | 48.58 | 9.91 | 0.20 |
|  | PL (mm) | 1093 | 156.79 | 23.93 | 0.15 |
|  | EW (g) | 1093 | 89.90 | 41.87 | 0.47 |
| 18-Mar | H25 (mm) | 748 | 57.01 | 11.79 | 0.21 |
|  | H50 (mm) | 748 | 66.95 | 14.81 | 0.22 |
|  | H75 (mm) | 748 | 54.08 | 13.25 | 0.24 |
|  | PL (mm) | 748 | 172.64 | 31.98 | 0.19 |
|  | EW (g) | 748 | 123.64 | 68.88 | 0.56 |
|  | ΔH25 | 743 | 6.06 | 6.07 | 1.00 |
|  | ΔH50 | 743 | 5.87 | 6.85 | 1.17 |
|  | ΔH75 | 743 | 4.01 | 6.91 | 1.72 |
|  | ΔPL | 743 | 12.55 | 14.00 | 1.12 |
|  | ΔEW | 743 | 28.44 | 34.41 | 1.21 |
| 18-Jun | H25 (mm) | 742 | 62.09 | 12.85 | 0.21 |
|  | H50 (mm) | 742 | 74.12 | 15.74 | 0.21 |
|  | H75 (mm) | 742 | 59.22 | 13.33 | 0.23 |
|  | PL (mm) | 742 | 186.81 | 34.32 | 0.18 |
|  | EW (g) | 742 | 156.22 | 85.26 | 0.55 |
|  | ΔH25 | 737 | 10.99 | 7.14 | 0.65 |
|  | ΔH50 | 737 | 12.84 | 7.80 | 0.61 |
|  | ΔH75 | 737 | 9.00 | 7.25 | 0.81 |
|  | ΔPL | 737 | 26.18 | 16.81 | 0.64 |
|  | ΔEW | 737 | 60.15 | 50.73 | 0.84 |
| 18-Oct | H25 (mm) | 719 | 66.94 | 13.52 | 0.20 |
|  | H50 (mm) | 719 | 79.99 | 16.40 | 0.21 |
|  | H75 (mm) | 719 | 61.26 | 12.70 | 0.21 |
|  | PL (mm) | 719 | 200.64 | 35.38 | 0.18 |
|  | EW (g) | 719 | 191.93 | 99.96 | 0.52 |
|  | ΔH25 | 715 | 15.85 | 7.80 | 0.49 |
|  | ΔH50 | 715 | 18.75 | 8.43 | 0.45 |
|  | ΔH75 | 715 | 11.08 | 6.78 | 0.61 |
|  | ΔPL | 715 | 40.03 | 18.02 | 0.45 |
|  | ΔEW | 715 | 95.83 | 64.50 | 0.67 |
| 19-Jan | H25 (mm) | 704 | 77.03 | 14.74 | 0.19 |
|  | H50 (mm) | 704 | 91.21 | 17.68 | 0.19 |
|  | H75 (mm) | 704 | 69.50 | 14.07 | 0.20 |
|  | PL (mm) | 704 | 228.87 | 38.95 | 0.17 |
|  | EW (g) | 704 | 282.36 | 135.04 | 0.48 |
|  | ΔH25 | 699 | 26.05 | 9.51 | 0.36 |
|  | ΔH50 | 699 | 30.05 | 10.62 | 0.35 |
|  | ΔH75 | 699 | 19.43 | 8.20 | 0.42 |
|  | ΔPL | 699 | 68.34 | 23.48 | 0.34 |
|  | ΔEW | 699 | 186.65 | 101.57 | 0.54 |
| 19-Apr | H25 (mm) | 698 | 84.29 | 15.35 | 0.18 |
|  | H50 (mm) | 698 | 98.29 | 17.85 | 0.18 |
|  | H75 (mm) | 698 | 75.85 | 14.32 | 0.19 |
|  | PL (mm) | 698 | 245.27 | 38.80 | 0.16 |
|  | EW (g) | 698 | 343.40 | 150.48 | 0.44 |
|  | ΔH25 | 693 | 33.25 | 10.64 | 0.32 |
|  | ΔH50 | 693 | 37.03 | 11.50 | 0.31 |
|  | ΔH75 | 693 | 25.67 | 8.93 | 0.35 |
|  | ΔPL | 693 | 84.58 | 24.82 | 0.29 |
|  | ΔEW | 693 | 247.52 | 118.42 | 0.48 |
| 19-Aug | H25 (mm) | 630 | 89.24 | 15.42 | 0.17 |
|  | H50 (mm) | 630 | 103.84 | 17.63 | 0.17 |
|  | H75 (mm) | 630 | 82.65 | 14.62 | 0.18 |
|  | PL (mm) | 630 | 256.25 | 38.77 | 0.15 |
|  | EW (g) | 630 | 389.10 | 163.00 | 0.42 |
|  | ΔH25 | 627 | 38.20 | 10.76 | 0.28 |
|  | ΔH50 | 627 | 42.67 | 11.33 | 0.27 |
|  | ΔH75 | 627 | 32.57 | 9.20 | 0.28 |
|  | ΔPL | 627 | 95.81 | 25.45 | 0.27 |
|  | ΔEW | 627 | 293.68 | 132.22 | 0.45 |
| 19-Nov | H25 (mm) | 694 | 90.10 | 15.71 | 0.17 |
|  | H50 (mm) | 694 | 105.53 | 18.67 | 0.18 |
|  | H75 (mm) | 694 | 85.63 | 15.81 | 0.18 |
|  | PL (mm) | 694 | 264.09 | 40.47 | 0.15 |
|  | EW (g) | 694 | 426.34 | 178.06 | 0.42 |
|  | ΔH25 | 689 | 39.08 | 11.22 | 0.29 |
|  | ΔH50 | 689 | 44.34 | 12.37 | 0.28 |
|  | ΔH75 | 689 | 35.54 | 10.20 | 0.29 |
|  | ΔPL | 689 | 103.59 | 27.33 | 0.26 |
|  | ΔEW | 689 | 330.78 | 147.23 | 0.45 |

Table S4: Heritability estimates (in bold), Pearson's phenotypic (above the diagonal) and genetic correlations (bellow the diagonal) in November 2017 (Nov17), March 2018 (Mar18), June 2018 (Jun18), October 2018 (Oct18), January 2019 (Jan19), April 2019 (Apr19), August 2019 (Aug19) and November 2019 (Nov19). Means ± standard deviations (SD) are indicated for height at 25% (H25), height at 50% (H50), height at 75% (H75), peduncle length (PL), estimated weight (EW), delta height 25% (ΔH25), delta height 50% (ΔH50), delta height 75% (ΔH75), delta peduncle length (ΔPL) and delta estimated weight (ΔEW). The last row in italic reports the additive genetic variance (vA) of each trait.

| **17-Nov** | **H25** | **H50** | **H75** | **PL** | **EW** |  |  |  |  |  |
| --- | --- | --- | --- | --- | --- | --- | --- | --- | --- | --- |
| H25 | **0.67±0.05** | 0.92±0.01 | 0.85±0.02 | 0.89±0.04 | 0.88±0.07 |  |  |  |  |  |
| H50 | 1.00±0.00 | **0.75±0.05** | 0.95±0.01 | 0.96±0.02 | 0.95±0.04 |  |  |  |  |  |
| H75 | 0.98±0.01 | 0.99±0.00 | **0.73±0.05** | 0.93±0.03 | 0.91±0.05 |  |  |  |  |  |
| PL | 1.00±0.00 | 0.98±0.01 | 0.94±0.01 | **0.74±0.05** | 0.99±0.01 |  |  |  |  |  |
| EW | 0.99±0.01 | 0.97±0.01 | 0.94±0.00 | 1.00±0.00 | **0.75±0.05** |  |  |  |  |  |
| *vA* | *25.89* | *29.23* | *24.8* | *149.87* | *458.59* |  |  |  |  |  |
|  |  |  |  |  |  |  |  |  |  |  |
| **18-Mar** | **H25** | **H50** | **H75** | **PL** | **EW** | **ΔH25** | **ΔH50** | **ΔH75** | **ΔPL** | **ΔEW** |
| H25 | **0.75±0.06** | 0.98±0.01 | 0.94±0.01 | 0.98±0.02 | 0.97±0.05 | 0.65±0.01 | 0.69±0.02 | 0.55±0.02 | 0.73±0.03 | 0.84±0.06 |
| H50 | 0.99±0.00 | **0.74±0.06** | 0.96±0.01 | 0.98±0.02 | 0.96±0.05 | 0.64±0.01 | 0.74±0.01 | 0.60±0.01 | 0.74±0.02 | 0.83±0.05 |
| H75 | 0.97±0.01 | 0.98±0.01 | **0.71±0.06** | 0.94±0.03 | 0.91±0.08 | 0.64±0.01 | 0.73±0.01 | 0.70±0.01 | 0.74±0.03 | 0.82±0.05 |
| PL | 0.99±0.00 | 0.99±0.00 | 0.96±0.01 | **0.76±0.06** | 0.98±0.02 | 0.62±0.01 | 0.68±0.01 | 0.55±0.01 | 0.74±0.01 | 0.84±0.02 |
| EW | 0.99±0.00 | 0.98±0.01 | 0.94±0.02 | 0.99±0.00 | **0.75±0.06** | 0.6±0.00 | 0.66±0.00 | 0.52±0.00 | 0.73±0.01 | 0.87±0.01 |
| ΔH25 | 0.83±0.07 | 0.86±0.06 | 0.89±0.06 | 0.83±0.07 | 0.78±0.08 | **0.28±0.07** | 0.76±0.03 | 0.58±0.03 | 0.69±0.06 | 0.69±0.15 |
| ΔH50 | 0.77±0.06 | 0.81±0.05 | 0.84±0.05 | 0.79±0.06 | 0.75±0.07 | 1.00±0.01 | **0.42±0.07** | 0.84±0.02 | 0.88±0.04 | 0.83±0.10 |
| ΔH75 | 0.60±0.10 | 0.65±0.09 | 0.71±0.08 | 0.59±0.10 | 0.53±0.11 | 1.00±0.03 | 0.99±0.02 | **0.35±0.08** | 0.77±0.05 | 0.70±0.13 |
| ΔPL | 0.83±0.05 | 0.86±0.05 | 0.88±0.04 | 0.84±0.05 | 0.80±0.06 | 1.00±0.02 | 1.00±0.01 | 0.97±0.05 | **0.46±0.08** | 0.95±0.03 |
| ΔEW | 0.93±0.02 | 0.93±0.02 | 0.93±0.03 | 0.93±0.02 | 0.93±0.02 | 0.96±0.03 | 0.95±0.03 | 0.84±0.07 | 0.95±0.01 | **0.56±0.07** |
| *vA* | *32.03* | *52.68* | *46.27* | *243.27* | *1159.21* | *24.37* | *24.26* | *29.48* | *98.7* | *477.98* |
|  |  |  |  |  |  |  |  |  |  |  |
| **18-Jun** | **H25** | **H50** | **H75** | **PL** | **EW** | **ΔH25** | **ΔH50** | **ΔH75** | **ΔPL** | **ΔEW** |
| H25 | **0.74±0.06** | 0.99±0.01 | 0.94±0.01 | 0.99±0.02 | 0.98±0.05 | 0.72±0.01 | 0.75±0.01 | 0.56±0.02 | 0.78±0.03 | 0.91±0.06 |
| H50 | 0.99±0.00 | **0.73±0.06** | 0.96±0.01 | 0.98±0.02 | 0.96±0.05 | 0.7±0.01 | 0.78±0.01 | 0.58±0.01 | 0.77±0.03 | 0.89±0.05 |
| H75 | 0.98±0.01 | 0.99±0.00 | **0.72±0.06** | 0.93±0.03 | 0.92±0.09 | 0.68±0.01 | 0.75±0.01 | 0.70±0.01 | 0.74±0.03 | 0.85±0.07 |
| PL | 1.00±0.00 | 0.99±0.00 | 0.97±0.01 | **0.69±0.07** | 0.98±0.02 | 0.70±0.01 | 0.74±0.01 | 0.54±0.01 | 0.78±0.01 | 0.91±0.02 |
| EW | 0.99±0.00 | 0.98±0.01 | 0.97±0.01 | 0.99±0.00 | **0.69±0.06** | 0.69±0.00 | 0.73±0.00 | 0.53±0.00 | 0.76±0.00 | 0.93±0.01 |
| ΔH25 | 0.87±0.05 | 0.85±0.05 | 0.86±0.06 | 0.87±0.05 | 0.84±0.06 | **0.37±0.08** | 0.81±0.02 | 0.59±0.03 | 0.79±0.05 | 0.78±0.17 |
| ΔH50 | 0.87±0.05 | 0.87±0.04 | 0.86±0.05 | 0.87±0.05 | 0.87±0.05 | 1.00±0.01 | **0.41±0.08** | 0.81±0.02 | 0.91±0.03 | 0.86±0.12 |
| ΔH75 | 0.75±0.09 | 0.76±0.08 | 0.77±0.08 | 0.76±0.09 | 0.75±0.09 | 0.97±0.04 | 0.98±0.02 | **0.27±0.07** | 0.72±0.06 | 0.66±0.19 |
| ΔPL | 0.85±0.05 | 0.83±0.05 | 0.83±0.05 | 0.85±0.05 | 0.85±0.05 | 0.99±0.01 | 0.97±0.02 | 0.93±0.05 | **0.41±0.08** | 0.93±0.04 |
| ΔEW | 0.95±0.02 | 0.93±0.02 | 0.93±0.02 | 0.95±0.02 | 0.96±0.01 | 0.95±0.03 | 0.95±0.02 | 0.86±0.07 | 0.96±0.02 | **0.57±0.07** |
| *vA* | *41.36* | *64.68* | *45.64* | *346.03* | *2126.6* | *29.73* | *32.1* | *33.25* | *149.41* | *997.8* |
|  |  |  |  |  |  |  |  |  |  |  |
| **18-Oct** | **H25** | **H50** | **H75** | **PL** | **EW** | **ΔH25** | **ΔH50** | **ΔH75** | **ΔPL** | **ΔEW** |
| H25 | **0.74±0.06** | 0.99±0.01 | 0.95±0.01 | 0.98±0.02 | 0.97±0.06 | 0.75±0.01 | 0.79±0.01 | 0.54±0.02 | 0.78±0.03 | 0.93±0.07 |
| H50 | 0.99±0.00 | **0.75±0.06** | 0.96±0.01 | 0.98±0.02 | 0.96±0.06 | 0.74±0.01 | 0.79±0.01 | 0.55±0.01 | 0.78±0.03 | 0.92±0.06 |
| H75 | 0.98±0.01 | 0.99±0.01 | **0.68±0.07** | 0.94±0.04 | 0.93±0.11 | 0.71±0.02 | 0.77±0.02 | 0.64±0.02 | 0.74±0.04 | 0.88±0.09 |
| PL | 0.99±0.00 | 0.99±0.00 | 0.97±0.01 | **0.7±0.06** | 0.98±0.02 | 0.72±0.01 | 0.77±0.01 | 0.53±0.01 | 0.79±0.01 | 0.94±0.02 |
| EW | 0.99±0.00 | 0.98±0.01 | 0.96±0.01 | 0.99±0.00 | **0.7±0.06** | 0.70±0.00 | 0.74±0.00 | 0.50±0.00 | 0.76±0.00 | 0.95±0.01 |
| ΔH25 | 0.86±0.05 | 0.85±0.05 | 0.85±0.06 | 0.85±0.05 | 0.83±0.06 | **0.46±0.08** | 0.86±0.02 | 0.64±0.02 | 0.81±0.05 | 0.78±0.19 |
| ΔH50 | 0.88±0.04 | 0.88±0.04 | 0.86±0.04 | 0.87±0.04 | 0.86±0.05 | 1.00±0.01 | **0.52±0.08** | 0.82±0.02 | 0.94±0.03 | 0.87±0.14 |
| ΔH75 | 0.66±0.11 | 0.65±0.10 | 0.67±0.1 | 0.66±0.11 | 0.64±0.11 | 0.94±0.04 | 0.90±0.05 | **0.28±0.07** | 0.75±0.07 | 0.64±0.27 |
| ΔPL | 0.85±0.05 | 0.83±0.05 | 0.81±0.06 | 0.85±0.05 | 0.83±0.05 | 0.99±0.01 | 0.98±0.01 | 0.88±0.06 | **0.47±0.08** | 0.91±0.05 |
| ΔEW | 0.96±0.01 | 0.95±0.01 | 0.93±0.02 | 0.96±0.01 | 0.97±0.01 | 0.93±0.03 | 0.94±0.03 | 0.75±0.09 | 0.94±0.02 | **0.63±0.07** |
| *vA* | *44.53* | *63.09* | *46.72* | *348.67* | *2791.05* | *31.19* | *31.57* | *32.3* | *156.94* | *1407.77* |
|  |  |  |  |  |  |  |  |  |  |  |
| **19-Jan** | **H25** | **H50** | **H75** | **PL** | **EW** | **ΔH25** | **ΔH50** | **ΔH75** | **ΔPL** | **ΔEW** |
| H25 | **0.730.06** | 0.98±0.01 | 0.96±0.01 | 0.98±0.02 | 0.97±0.09 | 0.80±0.01 | 0.79±0.02 | 0.68±0.02 | 0.80±0.04 | 0.94±0.09 |
| H50 | 0.99±0.00 | **0.72±0.06** | 0.97±0.01 | 0.98±0.02 | 0.96±0.08 | 0.80±0.01 | 0.82±0.01 | 0.71±0.01 | 0.80±0.03 | 0.94±0.08 |
| H75 | 0.97±0.01 | 0.99±0.00 | **0.67±0.07** | 0.95±0.03 | 0.94±0.13 | 0.76±0.02 | 0.79±0.02 | 0.74±0.01 | 0.78±0.04 | 0.91±0.11 |
| PL | 0.99±0.00 | 0.98±0.00 | 0.96±0.01 | **0.7±0.07** | 0.98±0.03 | 0.78±0.01 | 0.79±0.01 | 0.68±0.01 | 0.82±0.01 | 0.96±0.03 |
| EW | 0.98±0.01 | 0.97±0.01 | 0.95±0.01 | 0.99±0.00 | **0.71±0.06** | 0.75±0.00 | 0.75±0.00 | 0.64±0.00 | 0.78±0.00 | 0.97±0.01 |
| ΔH25 | 0.88±0.04 | 0.87±0.04 | 0.82±0.05 | 0.84±0.04 | 0.80±0.05 | **0.54±0.08** | 0.90±0.02 | 0.77±0.02 | 0.87±0.05 | 0.82±0.23 |
| ΔH50 | 0.90±0.04 | 0.90±0.03 | 0.85±0.04 | 0.87±0.04 | 0.84±0.05 | 1.00±0.00 | **0.5±0.08** | 0.90±0.01 | 0.95±0.03 | 0.85±0.19 |
| ΔH75 | 0.90±0.06 | 0.90±0.05 | 0.86±0.06 | 0.88±0.06 | 0.85±0.07 | 1.00±0.01 | 0.99±0.02 | **0.32±0.08** | 0.87±0.05 | 0.75±0.31 |
| ΔPL | 0.91±0.04 | 0.89±0.04 | 0.85±0.05 | 0.9±0.04 | 0.88±0.04 | 0.98±0.01 | 0.98±0.01 | 0.99±0.01 | **0.46±0.08** | 0.89±0.07 |
| ΔEW | 0.98±0.01 | 0.96±0.01 | 0.94±0.02 | 0.98±0.01 | 0.99±0.00 | 0.86±0.04 | 0.90±0.03 | 0.91±0.05 | 0.93±0.02 | **0.64±0.07** |
| *vA* | *56.37* | *84.17* | *61.28* | *440.36* | *5100.25* | *41.43* | *56.06* | *45.67* | *287.91* | *3507.55* |
|  |  |  |  |  |  |  |  |  |  |  |
| **19-Apr** | **H25** | **H50** | **H75** | **PL** | **EW** | **ΔH25** | **ΔH50** | **ΔH75** | **ΔPL** | **ΔEW** |
| H25 | **0.72±0.06** | 0.99±0.01 | 0.95±0.01 | 0.98±0.02 | 0.97±0.09 | 0.81±0.02 | 0.80±0.02 | 0.69±0.02 | 0.79±0.04 | 0.96±0.09 |
| H50 | 1.00±0.00 | **0.72±0.06** | 0.97±0.01 | 0.98±0.02 | 0.96±0.09 | 0.81±0.01 | 0.81±0.01 | 0.71±0.01 | 0.80±0.03 | 0.95±0.08 |
| H75 | 0.97±0.01 | 0.98±0.01 | **0.68±0.07** | 0.95±0.03 | 0.94±0.14 | 0.77±0.02 | 0.78±0.02 | 0.75±0.02 | 0.76±0.04 | 0.92±0.12 |
| PL | 0.99±0.00 | 0.98±0.00 | 0.96±0.01 | **0.7±0.07** | 0.98±0.03 | 0.79±0.01 | 0.78±0.01 | 0.69±0.01 | 0.81±0.01 | 0.97±0.03 |
| EW | 0.98±0.01 | 0.98±0.01 | 0.95±0.01 | 0.99±0.00 | **0.72±0.06** | 0.75±0.00 | 0.74±0.00 | 0.65±0.00 | 0.76±0.00 | 0.98±0.01 |
| ΔH25 | 0.89±0.04 | 0.88±0.04 | 0.84±0.05 | 0.86±0.04 | 0.82±0.05 | **0.52±0.08** | 0.91±0.02 | 0.79±0.02 | 0.89±0.04 | 0.83±0.24 |
| ΔH50 | 0.90±0.04 | 0.89±0.04 | 0.86±0.05 | 0.88±0.04 | 0.87±0.05 | 0.97±0.04 | **0.48±0.08** | 0.91±0.01 | 0.95±0.02 | 0.84±0.21 |
| ΔH75 | 0.82±0.06 | 0.82±0.06 | 0.82±0.06 | 0.81±0.07 | 0.78±0.07 | 0.98±0.02 | 0.96±0.02 | **0.38±0.08** | 0.88±0.05 | 0.75±0.33 |
| ΔPL | 0.89±0.04 | 0.87±0.04 | 0.84±0.05 | 0.89±0.04 | 0.87±0.04 | 0.98±0.01 | 0.98±0.01 | 0.96±0.02 | **0.46±0.08** | 0.87±0.09 |
| ΔEW | 0.98±0.01 | 0.97±0.01 | 0.95±0.01 | 0.99±0.00 | 0.99±0.00 | 0.88±0.04 | 0.92±0.03 | 0.84±0.06 | 0.93±0.03 | **0.66±0.07** |
| *vA* | *65.4* | *90.5* | *64.27* | *450.7* | *6379.85* | *57* | *71.92* | *51.43* | *335.86* | *4711.91* |
|  |  |  |  |  |  |  |  |  |  |  |
| **19-Aug** | **H25** | **H50** | **H75** | **PL** | **EW** | **ΔH25** | **ΔH50** | **ΔH75** | **ΔPL** | **ΔEW** |
| H25 | **0.7±0.07** | 0.99±0.01 | 0.93±0.01 | 0.98±0.02 | 0.96±0.11 | 0.83±0.02 | 0.81±0.02 | 0.70±0.02 | 0.80±0.04 | 0.95±0.10 |
| H50 | 0.99±0.00 | **0.71±0.07** | 0.96±0.01 | 0.97±0.02 | 0.96±0.11 | 0.81±0.01 | 0.82±0.01 | 0.73±0.01 | 0.79±0.04 | 0.94±0.10 |
| H75 | 0.97±0.01 | 0.98±0.01 | **0.69±0.07** | 0.92±0.04 | 0.91±0.18 | 0.74±0.02 | 0.77±0.02 | 0.78±0.02 | 0.73±0.05 | 0.90±0.16 |
| PL | 0.99±0.00 | 0.98±0.01 | 0.95±0.01 | **0.69±0.07** | 0.98±0.03 | 0.80±0.01 | 0.80±0.01 | 0.70±0.01 | 0.82±0.02 | 0.97±0.03 |
| EW | 0.98±0.01 | 0.97±0.01 | 0.95±0.02 | 0.99±0.00 | **0.72±0.07** | 0.76±0.00 | 0.75±0.00 | 0.66±0.00 | 0.77±0.00 | 0.98±0.01 |
| ΔH25 | 0.90±0.03 | 0.88±0.04 | 0.86±0.05 | 0.88±0.04 | 0.86±0.05 | **0.48±0.08** | 0.92±0.02 | 0.77±0.02 | 0.89±0.04 | 0.83±0.27 |
| ΔH50 | 0.90±0.04 | 0.89±0.04 | 0.87±0.05 | 0.89±0.04 | 0.87±0.05 | 1.00±0.00 | **0.48±0.08** | 0.90±0.01 | 0.95±0.03 | 0.84±0.25 |
| ΔH75 | 0.85±0.06 | 0.85±0.05 | 0.84±0.05 | 0.84±0.06 | 0.82±0.07 | 0.98±0.02 | 0.98±0.01 | **0.41±0.08** | 0.85±0.06 | 0.74±0.39 |
| ΔPL | 0.86±0.04 | 0.83±0.05 | 0.80±0.06 | 0.87±0.04 | 0.85±0.05 | 0.98±0.01 | 0.98±0.01 | 0.96±0.02 | **0.5±0.08** | 0.87±0.10 |
| ΔEW | 0.97±0.01 | 0.96±0.01 | 0.94±0.02 | 0.99±0.00 | 0.99±0.00 | 0.91±0.03 | 0.92±0.03 | 0.87±0.05 | 0.92±0.03 | **0.68±0.07** |
| *vA* | *71.69* | *93.33* | *67.95* | *471.74* | *7713.83* | *62.68* | *70.63* | *53.42* | *339.33* | *5792.1* |
|  |  |  |  |  |  |  |  |  |  |  |
| **19-Nov** | **H25** | **H50** | **H75** | **PL** | **EW** | **ΔH25** | **ΔH50** | **ΔH75** | **ΔPL** | **ΔEW** |
| H25 | **0.73±0.06** | 0.99±0.01 | 0.95±0.01 | 0.98±0.02 | 0.97±0.11 | 0.82±0.02 | 0.82±0.02 | 0.75±0.02 | 0.79±0.04 | 0.95±0.11 |
| H50 | 0.99±0.00 | **0.72±0.06** | 0.97±0.01 | 0.97±0.02 | 0.96±0.10 | 0.81±0.01 | 0.83±0.01 | 0.77±0.01 | 0.79±0.03 | 0.95±0.10 |
| H75 | 0.95±0.01 | 0.98±0.01 | **0.68±0.07** | 0.94±0.03 | 0.93±0.16 | 0.75±0.02 | 0.78±0.02 | 0.80±0.01 | 0.75±0.04 | 0.91±0.15 |
| PL | 0.99±0.00 | 0.98±0.00 | 0.94±0.01 | **0.69±0.07** | 0.98±0.03 | 0.80±0.01 | 0.81±0.01 | 0.76±0.01 | 0.82±0.01 | 0.97±0.03 |
| EW | 0.98±0.01 | 0.97±0.01 | 0.93±0.02 | 1.00±0.00 | **0.72±0.06** | 0.76±0.00 | 0.77±0.00 | 0.72±0.00 | 0.78±0.00 | 0.98±0.01 |
| ΔH25 | 0.89±0.03 | 0.88±0.04 | 0.82±0.05 | 0.89±0.04 | 0.86±0.04 | **0.56±0.08** | 0.92±0.02 | 0.81±0.02 | 0.89±0.04 | 0.83±0.28 |
| ΔH50 | 0.88±0.04 | 0.88±0.03 | 0.84±0.04 | 0.88±0.04 | 0.87±0.04 | 1.00±0.00 | **0.54±0.08** | 0.92±0.01 | 0.96±0.02 | 0.85±0.24 |
| ΔH75 | 0.88±0.05 | 0.89±0.05 | 0.86±0.05 | 0.88±0.05 | 0.86±0.06 | 0.97±0.05 | 0.99±0.01 | **0.43±0.08** | 0.89±0.05 | 0.79±0.33 |
| ΔPL | 0.85±0.04 | 0.83±0.04 | 0.77±0.06 | 0.86±0.04 | 0.86±0.04 | 0.99±0.01 | 0.98±0.01 | 0.95±0.02 | **0.51±0.08** | 0.87±0.10 |
| ΔEW | 0.97±0.01 | 0.96±0.01 | 0.91±0.02 | 0.99±0.00 | 0.99±0.00 | 0.92±0.03 | 0.92±0.03 | 0.90±0.05 | 0.92±0.03 | **0.68±0.07** |
| *vA* | *66.05* | *98.03* | *78.91* | *524.09* | *9178.11* | *59.67* | *74.68* | *63.01* | *388.12* | *7194.48* |
